# When Are Dopant *d*‑States Free-Atom-Like? Periodic Trends and Confinement Effects in Single-Atom Alloys

**DOI:** 10.1021/jacs.5c07771

**Published:** 2025-10-03

**Authors:** Fabian Berger, Angelos Michaelides

**Affiliations:** Yusuf Hamied Department of Chemistry, 2152 University of Cambridge, Cambridge CB2 1EW, U.K.

## Abstract

The free-atom-like character of dopant *d*-states is a defining feature of single-atom alloys (SAAs), contributing to their exceptional selectivity and activity in heterogeneous catalysis. Based on reliable density functional theory (DFT) calculations for the full series of 4*d* transition metal (TM) dopants embedded in various hosts, we provide a unified perspective on when dopants exhibit this characteristic and how it can be tuned. Only late TM dopants exhibit the narrow, free-atom-like *d*-bands typically associated with SAAs, whereas early TM dopants display significantly broader *d*-bands that approach the width of host metal *d*-bands. This variation is not driven by increased dopant–host *d*-state mixing, which remains minimal across the series. Instead, we attribute the observed periodic trend to differences in the spatial extent of the localized dopant states and their overlap with surrounding host atoms, as well as to avoided hybridization associated with *d*-state filling. We further corroborate that dopant confinement, quantified by the number and proximity of surrounding host atoms, is as a key factor: more confined dopants exhibit broader *d*-bands, whereas less confined dopants feature narrower *d*-bands. Reduced dopant confinement also stabilizes high-spin configurations, enhancing spin polarization for certain 4*d* elements. Together, these findings offer fundamental insights into the origins of the unique electronic structure of SAAs. Building on these findings, we establish design principles for tuning dopant *d*-band shape and spin and illustrate how such modifications impact catalytic selectivity. The developed guidelines are also encapsulated in a machine learning model that predicts *d*-band widths, facilitating the rational design of SAAs.

## Graphical abstract

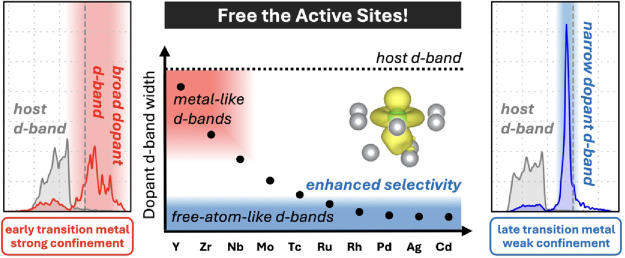

## Introduction

Heterogeneous catalysis is essential for most industrial processes and has been a major driver of societal prosperity. The performance of a catalyst is mainly defined by its activity and selectivity. Activity refers to its ability to lower reaction barriers, allowing reactions to proceed at lower temperatures or pressures, thereby reducing costs. Selectivity ensures that the desired products form consistently over time with minimal interference from side reactions, byproducts, or catalyst poisoning. Transition metals (TMs) are widely used as solid catalysts and have been highly successful in heterogeneous catalysis. However, they typically follow linear scaling relations between barrier heights and adsorption strength,^1,2^ as described by the Bro̷nsted–Evans–Polanyi (BEP) principle.^3–6^ This leads to the well-known activity-selectivity trade-off and the Sabatier principle.^7,8^ Catalysts can be too unreactive but also too reactive. While the product formation is rate-determining for less active catalysts, more active catalysts tend to bind reaction products more strongly, hindering their release.

Single-atom alloys^9–15^ (SAAs) are a relatively new class of materials composed of a chemically rather inert host metal, typically copper, silver, or gold, and atomically dispersed transition metal dopants that serve as active sites for catalytic reactions. They have gained attention for their ability to circumvent the activity-selectivity trade-off, breaking long-standing linear scaling relations.^16–20^ They have already been successfully applied in selective hydrogenation,^12,21^ selective oxidation,^22^ dehydrogenation,^23^ and CO oxidation,^24^ and have enormous potential in heterogeneous catalysis in general.

The composition of SAAs gives rise to two key features that define their unique catalytic properties. First, SAAs exhibit bifunctionality:^9^ dopant atoms form catalytically active sites that are separated from the more inert host surface, which can act as a desorption site for reaction products. While the dopant controls the activity of the catalyst, the host can help suppress side reactions once products have left the active site and spilled over onto the host surface.^25^ This spatial and functional separation has been associated with enhanced selectivity by facilitating product desorption and reducing unwanted side reactions.

The second key feature responsible for the enhanced selectivity of SAAs is the free-atom-like nature of dopant *d*-states, a connection that has been widely discussed.^26–32^ At the active site, dopant *d*-bands which are narrow in energy interact strongly with reactant molecular orbitals only when their energies align,^29,33^ a concept more commonly encountered in homogeneous catalysis, where the narrow electronic states of molecular complexes drive high selectivity. This behavior contrasts with the broader *d*-bands typical of metallic surfaces, which can interact with a wider range of reactants possessing more varied orbital energies.^34,35^ Beyond enabling greater selectivity through stricter energy matching, narrow *d*-bands can also strengthen adsorption when orbital energies do align.^29,33^ According to the Newns–Anderson–Grimley model,^26,29,36–39^ such narrow *d*-bands cause more pronounced splitting into bonding and antibonding states upon interaction with molecular orbitals. In contrast, broader, metal-like *d*-bands tend to cause broadening rather than distinct energy level splitting.

The free-atom-like character of the dopant’s *d*-states in SAAs was first reported in 2018,^26^ with earlier work noting band narrowing in a silver–rhodium bimetallic alloy.^28,40^ Since then, this free-atom-like nature has been used to explain the catalytic properties of SAAs^27,29^ and extended to alloys beyond the dilute limit.^30^ It has become a widely accepted and defining characteristic of SAAs.^41^ In this work, we provide a unified and holistic perspective on the extent to which dopant *d*-bands in SAAs exhibit a free-atom-like character and the conditions under which this beneficial property can be achieved.

While many excellent experimental and computational studies have been conducted on SAAs,^9,15,41–47^ a cohesive perspective that synthesizes these findings, clarifies under which conditions dopant states exhibit free-atom-like character, and shows how to translate this behavior into practical design rules is not yet established. Although most previous studies have not directly addressed this issue, they have provided indications in both directions. Some describe only certain SAAs as exhibiting free-atom-like behavior, implicitly suggesting that others may not.^27,29,30^ Others have explicitly noted that only a minority of SAAs display this characteristic,^26^ before focusing on those specific systems rather than exploring broader periodic trends. Conversely, some reports mention that ‘a sharp feature near the Fermi level was observed for most SAAs’,^15^ implying that the free-atom-like character may be more widespread. These, at first glance, seemingly misaligned observations highlight the need to integrate the separate findings into a coherent picture.

The only commonly cited constraint is that dopants must be atomically dispersed, without direct contact with other dopant atoms.^30^ Observations in bimetallic alloys outside the dilute limit further indicate that ‘most of the elements with localized *d*-states are late transition metals’,^30^ which aligns with trends compiled from other studies.^29,48^ From the current body of literature, it is clear that localized, free-atom-like *d*-states at active sites in SAAs are desirable. At the same time, there is no unified perspective on this behavior or the conditions under which it arises. Bringing clarity and consensus to these disparate observations is a primary motivation of this work.

## Discussion

In this work, we investigate the extent to which dopant *d*-states exhibit free-atom-like character across the 4*d* TM series. We perform density functional theory (DFT) calculations using the optB86b-vdW functional,^49^ with full computational details provided in Section S1 of the Supporting Information. Our study explores periodic trends in the dopant *d*-band width, examining the influence of both the dopant identity and the degree of confinement imposed by different host environments, as illustrated schematically in Figure 1.

**Figure 1:**
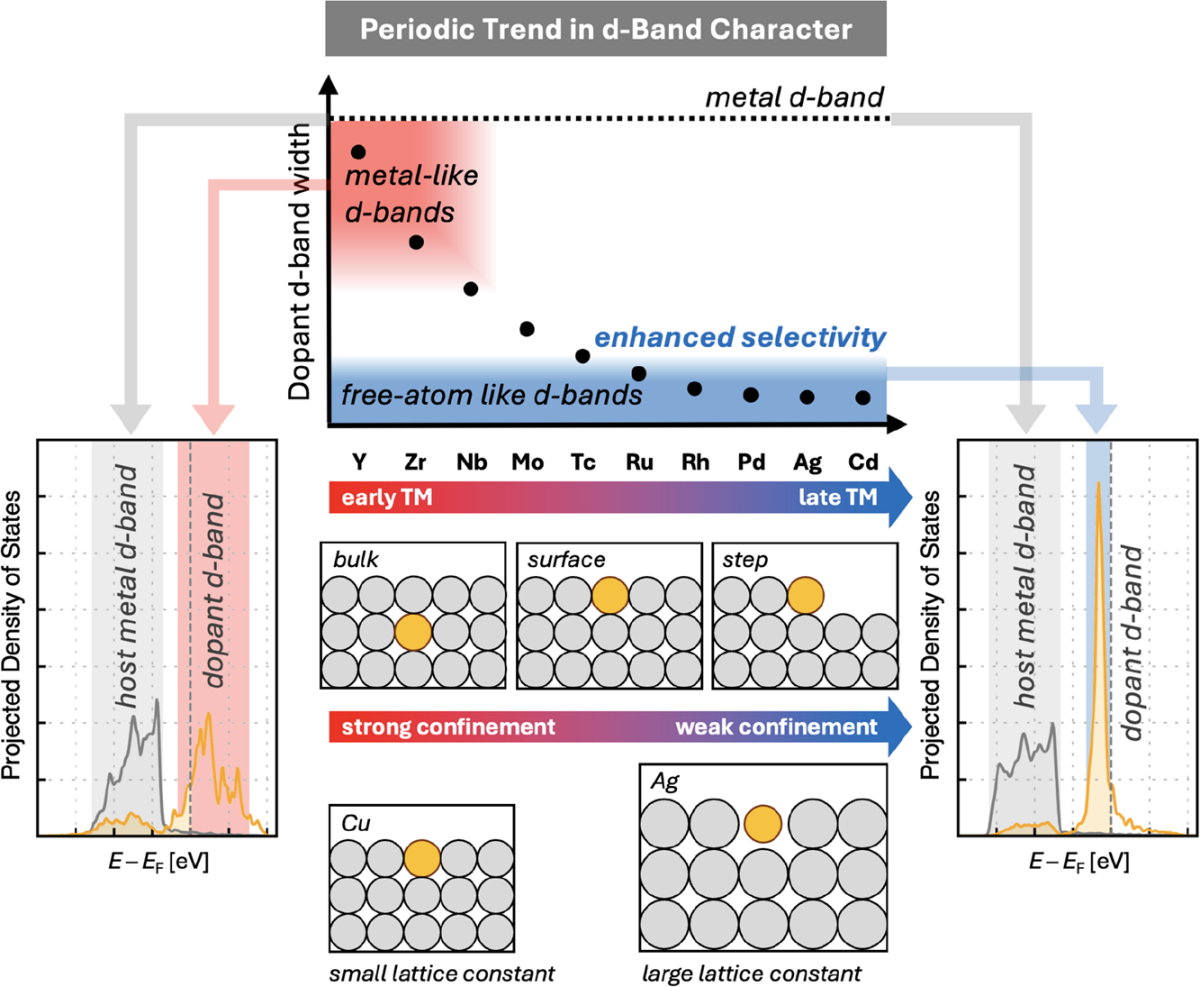
Schematic illustration of periodic trends in dopant *d*-band width (black circles) in SAAs. Early TM dopants exhibit broad *d*-bands resembling those of bulk metals (red-shaded area), while late TM dopants feature narrow, free-atom-like *d*-bands (blue-shaded area). Representative projected densities of states, normalized per atom, are shown for SAAs with a broad (left) and a narrow (right) dopant *d*-band. Dopant *d*-bands are shown in orange, and host metal *d*-bands in gray. Sketches are illustrative only; all calculations use 3 × 3 × 6 slab models.

We consider the dopants Y, Zr, Nb, Mo, Tc, Ru, Rh, and Pd embedded in bulk and in the (111) and (100) surface facets, as well as the step edge of (211) surfaces of Cu and Ag. This selection of systems allows us to probe how the electronic structure of the dopants is influenced by confinement, considered here as a combined effect of the number of host atoms directly coordinated to the dopant and the distance between the dopant and its surrounding host atoms. The use of Cu and Ag hosts enables comparison between more (Cu) and less (Ag) confining environments, owing to their differing lattice constants (3.60 Å vs 4.09 Å) and interatomic distances (2.55 Å vs 2.89 Å). To complete the 4*d* TM series, we also include Ag and Cd dopants; however, since their *d*-states lie within or below those of the host surfaces, rendering them less relevant for SAA reactivity, these results are reported in Section S2 of the Supporting Information. While this study provides insights into SAAs containing all 4*d* TMs, it should be noted that some of these elements may exhibit a tendency to aggregate.^50^ However, our focus lies on the electronic structure of isolated dopants in the dilute limit, which defines the active site and underpins the catalytic properties of SAAs.

Before discussing the electronic structure of these systems in detail, we first clarify what we mean by free-atom-like character, a term that has been used with slightly varying interpretations. This character can be considered in two ways: (i) energetically narrow *d*-bands, with widths reminiscent of the discrete energy levels of free atoms, and (ii) spatially localized dopant *d*-states that resemble atomic orbitals in shape. As we will show, only the energetic aspect, specifically, the width of the dopant *d*-band in the projected density of states (pDOS), serves as a meaningful metric for assessing the extent of free-atom-like character. Periodic trends in dopant *d*-band widths, quantified as the full width at half-maximum (fwhm) of the main peak, along with the size and shape of the *d*-states for a representative early and late TM dopant, are illustrated in Figure 2.

**Figure 2:**
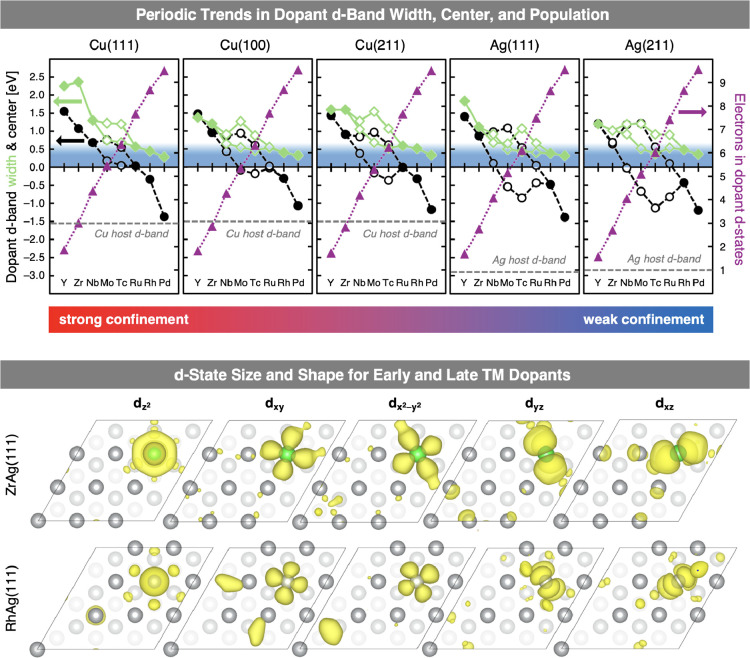
Top: Periodic trends in the electronic structure of 4*d* transition metal (TM) dopants embedded in host metal surfaces. The width of the main dopant *d*-band feature, measured as the full width at half-maximum (fwhm) in eV (green diamonds), decreases across the series, from early TMs with broad, metal-like *d*-bands to late TMs with narrow, free-atom-like *d*-bands. The center of the dopant *d*-band (black circles) shifts downward, approaching the upper edge of the host *d*-band (horizontal gray dashed lines) from early to late TMs. This trend is accompanied by increasing *d*-state population, shown as the sum of alpha and beta electrons (purple triangles). Filled symbols indicate low-spin states; open symbols represent the individual alpha and beta contributions in cases where a high-spin state at the dopant is more favorable. Blue-shaded regions highlight narrow, free-atom-like *d*-bands. Connecting lines are for illustrative purposes only. Bottom: Electron density isosurfaces (yellow) of selected Kohn–Sham states illustrate the orbital character and spatial extent of dopant *d*-states. The early TM dopant Zr (green atom), embedded in an Ag(111) surface (gray atoms), exhibits more spatially extended, atomic orbital-like states than the more compact *d*-states of the late TM dopant Rh (green atom). Surface atoms are shaded by depth: top layer (dark gray), second layer (medium gray), and third layer (light gray).

From Figure 2, two key trends emerge, corroborating prior work. First, the dopant *d*-band width decreases across the 4*d* series,^27,29,30,41^ from broad, metal-like *d*-bands for early TMs to narrow, free-atom-like *d*-bands for late TMs. Second, early TM dopants exhibit a pronounced sensitivity to their environment, with *d*-bands narrowing substantially in less confining hosts,^20,30,41,51^ while late TM dopants exhibit smaller changes. For instance, the Zr *d*-band narrows from 2.41 eV in bulk Cu, the most confining environment, to 1.02 eV at the step edge of Ag(211), the least confining surface.

Having established the two main trends in dopant *d*-band width, we now explore their underlying origins. A key requirement for narrow *d*-bands is weak interaction between the dopant and its environment—that is, electronic decoupling from the surrounding host metal. This behavior has also been experimentally observed, for example, in Cu dopants embedded in Ag surfaces, where narrow *d*-bands were reported,^26^ supporting our computational findings. When dopants are electronically decoupled, the electron distributions of their *d*-states closely resemble the atomic orbitals of an isolated atom, as if surrounded by vacuum, giving rise to the term ‘free-atom-like’. These spatially localized states are consistently observed across the entire series of investigated dopants, from early to late TMs. Such localized states stand in stark contrast to the strongly delocalized electronic states characteristic of bulk metals. Thus, while the localized nature of dopant *d*-states is necessary for the unique electronic structure of SAAs, it alone cannot explain the observed variation in dopant *d*-band widths.

The coupling strength between dopant and host states is generally understood to be governed by two key factors: the spatial overlap of the states and their energetic alignment. These considerations are also invoked in the SAA literature to rationalize state mixing and dopant *d*-band widths.^26,41^ Based on the energetic alignment argument, one would expect the dopant *d*-bands to broaden as we move from early to late TM dopants, with the dopant *d*-states approaching the host metal *d*-band from above. Our analysis, however, reveals the opposite trend. The main feature of the dopant *d*-band (green solid lines in Figure 2) becomes narrower as its center (black dashed lines) shifts closer in energy to the host *d*-band (horizontal gray dashed line). This finding demonstrates that, unless dopant and host *d*-bands directly overlap, energetic proximity does not drive substantial hybridization and therefore does not determine the dopant *d*-band width. Instead, our results corroborate that spatial confinement is the dominant factor and highlight orbital occupancy as an additional key contribution, particularly critical for the narrow *d*-bands of late TM dopants. That said, there are subtle indications of increased dopant–host interaction for late TM dopants. As shown in Figure 2, the *d*-states of Rh exhibit small but noticeably more delocalization into the host surface compared to those of Zr. Additionally, the pDOSs shown in Figures S1–S10 in the Supporting Information reveal minor contributions of dopant *d*-states within the energy range of the host metal *d*-band. These contributions, which are indicative of weak state mixing, are slightly more pronounced for late TM dopants. Nevertheless, despite these signs of slightly increased dopant–host interaction for late TM dopants, all dopant elements whose *d*-states do not directly overlap with those of the host remain largely electronically decoupled.

In addition to energetic misalignment, we suggest that the filling of the dopant (purple dotted lines in Figure 2) and host *d*-states could play a role in limiting state mixing; especially for late TM dopants that are in energetic proximity to the host *d*-band. The dopant *d*-state filling increases across the series, transitioning from mostly empty for early TMs to fully populated for late TMs. Similarly, the *d*-states of late coinage metal hosts are fully filled. Within a simple molecular orbital (MO) framework, hybridization between dopant and host orbitals would results in the formation of both bonding and antibonding states. As the number of dopant *d*-electrons increases from early to late TMs, a larger portion of electrons would occupy the destabilized antibonding states, rendering hybridization increasingly unfavorable. This new explanation expands the prevailing view that state mixing is governed primarily by energetic proximity and spatial overlap.^26,29^ While an energy mismatch between dopant and host *d*-states prevents mixing, the dopant’s *d*-electron count also plays a role. A schematic illustrating this concept is provided in Figure S11 in Section S3 in the Supporting Information.

Beyond the electronic structure, periodic trends in dopant–host interactions also manifest in the surface morphology of SAAs. As discussed in Section S4 in the Supporting Information, the height of the dopant relative to the host surface follows a clear periodic trend that primarily correlates with atomic size: very early and very late TM dopants protrude more strongly, with Y and Cd in Cu(111) protruding by 92 and 72 pm, respectively. In contrast, central dopants can even sit below the surface, such as Ru in Ag(111), which is recessed by 20 pm. While this periodic trend is largely dictated by dopant size, there appears to be a secondary effect: late TMs protrude more than early TMs of similar size. For example, the late TM Ag with an atomic radius of 145 pm protrudes by 30 pm when embedded in Cu(111), whereas the similarly sized early TM Nb with an atomic radius of 143 pm protrudes by only 7 pm on the same surface. We suggest that this asymmetry arises from suppressed state mixing for late TMs, which weakens dopant–host bonding and permits greater vertical relaxation, thereby accommodating size mismatch between the dopants and the host lattice. Minor interactions involving *s*- or *p*-states may also help retain these weakly bound dopants within the host surface,^15,50^ stabilizing the SAAs.

Importantly, we do not attribute the increased *d*-band width observed for early TM dopants to stronger dopant–host state mixing. In fact, as discussed above, early TMs are electronically more decoupled from the host than late TMs. Rather, the broader *d*-bands originate from intrinsic properties of the dopants. Across the TM series, the effective nuclear charge increases from left to right, leading to a progressive contraction of the *d*-orbitals. As a result, early TMs, which possess a lower effective nuclear charge, exhibit more diffuse and spatially extended *d*-states, and correspondingly larger atomic radii. As shown in Figure 2, this greater spatial extent leads to increased overlap with surrounding host atoms. This enhanced overlap amplifies the sensitivity of the dopant *d*-states to the local environment, resulting in more pronounced state-specific stabilization and destabilization. These stronger variations in the energy of individual *d*-states, which together form the dopant *d*-band, ultimately account for the broader *d*-band widths observed for early TMs.

*Building on these fundamental insights into the electronic structure of active dopant sites, we propose a practical design principle: reducing dopant confinement weakens state-specific interactions and creates a more isotropic environment. A tangible way to achieve this is by lowering the number of neighboring host atoms or increasing dopant–host distances through the choice of host metals or surface facets. This approach offers a pathway to tune early TM dopants toward the free-atom-like d-band character typically seen only in late TMs, expanding the range of SAAs with desirable catalytic properties.*

Figure 3 presents the pDOSs for the early TM dopant Zr and the late TM dopant Rh embedded in all investigated environments. These systems are selected to systematically vary the coordination environment of the dopants. In bulk Cu, the dopant is coordinated by 12 atoms (coordination number, *CN* = 12), while this number decreases to 9 in the (111) surface, 8 in the (100) surface, and 7 at the step-edge of a (211) surface. In addition to the coordination number, the nature of the host metal also influences dopant confinement. Cu is less inert than Ag, possessing a *d*-band that is higher in energy, which can lead to stronger interactions with dopants. Cu also has a smaller lattice constant (3.60 Å) compared to Ag (4.09 Å), resulting in shorter Cu–dopant distances and therefore stronger dopant confinement in Cu-based hosts. To quantify dopant confinement, we define two metrics that account for both the number and proximity of neighboring host atoms: a continuous coordination number and a discrete, volume-normalized coordination number, as described in Section S5 in the Supporting Information. While there is no unique expression for characterizing dopant confinement, both definitions yield the same ordering of decreasing dopant confinement:

Cubulk>Cu(111)>Cu(100)>Cu(211)>Ag(111)>Ag(211)

**Figure 3:**
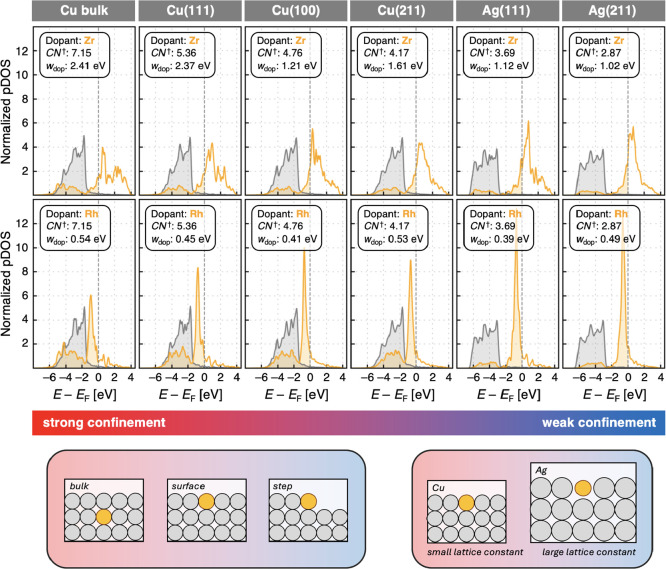
Projected densities of states (pDOSs) for the dopant atoms Zr and Rh (orange) and the Cu and Ag host surfaces (gray), normalized per atom, show that the width of the main feature of the *d*-bands, measured as full width at half-maximum (*w*_dop_), is broad for the early TM dopant Zr and narrow for the late TM dopant Rh. The *d*-bands of the host surfaces remain largely unchanged across the investigated systems, which can be ordered by decreasing dopant confinement as Cu-bulk, Cu(111), Cu(100), Cu(211), Ag(111), and Ag(211). This trend is illustrated by the corresponding continuous coordination number (*CN* ^†^), as described in detail in Section S5 in the Supporting Information. The dopant *d*-bands tend to narrow as confinement decreases, an effect that is particularly pronounced for early TM dopants with broader *d*-bands. The population of the *d*-bands up to the Fermi level (*E*_F_, gray dashed vertical line) is indicated by the colored filling of the enclosed curves. Confinement is illustrated schematically: a dopant atom (orange) in the bulk (gray atoms) experiences strong confinement; confinement decreases at the surface, and is lowest at a step edge. Similarly, host metals with larger lattice constants (Ag) are less confining than those with smaller lattice constants (Cu). Sketches are illustrative only; all calculations use 3 × 3 × 6 slab models.

In agreement with previous studies,^30,41,51–54^ varying the confinement leads to significant changes in dopant *d*-band width, with broader *d*-bands observed in more confined environments. Early TM dopants are more sensitive to their surroundings, showing greater variations in *d*-band width, whereas late TM dopants exhibit a weaker response to changing confinement. For example, in bulk Cu (the most confining system), the Zr *d*-band width is 2.41 eV, while at the step edge of Ag(211) (the least confining system), it narrows to 1.02 eV, constituting a substantial reduction of 1.4 eV. Late TM dopants show only a small change in *d*-band width but experience a noticeable change in the height of the main peak. This change is likely due to an incomplete decoupling of dopant *d*-states from Cu host metal states, whereas the dopant *d*-states are almost entirely decoupled from the host in Ag surfaces. The less coupling between dopant and host surface, the higher the main feature of the dopant *d*-band for late TMs.

To further disentangle the effects of local symmetry and dopant–host distance on the electronic structure of the active site, we calculate the *d*-band width of Zr embedded in Cu surface facets with increased lattice constants, up to that of Ag, and compare the results to the corresponding Ag surfaces. As the Cu lattice constant, and thus the dopant–host distance, increases, the dopant *d*-band narrows and approaches the width observed in the Ag host. This confirms that expanding the space available for the dopant *d*-electrons reduces the energetic splitting of the *d*-states. While this trend holds across all surface facets, the absolute *d*-band widths follow a facet-dependent order. This symmetry-driven effect, however, diminishes as dopant–host distances increase. To ensure that these conclusions are not biased by finite-size effects, we also tested larger 5 × 5 × 6 supercells for representative dopants and found that the resulting variations in *d*-band widths, centers, and occupations are minor compared to the periodic trends discussed here. Further details are provided in Section S6 in the Supporting Information.

Having established a comprehensive understanding of the periodic trends in the electronic structures of dopants, explored their fundamental origins, and proposed practical strategies to tailor *d*-band characteristics, we now demonstrate the broad applicability of these insights through a machine-learning (ML) model. The simple model developed predicts dopant *d*-band widths from physically grounded descriptors derived from our design guidelines: the atomic size of the dopant and host, the number of host atoms directly coordinating the dopant, and the formal *d*-electron count of the dopant. All of these are tabulated inputs that require no DFT calculations. Model details and further discussion are provided in Section S7 of the Supporting Information. The ML model captures 92% of the variance in dopant *d*-band width across all studied SAAs, with a root-mean-square deviation (RMSD) of 0.17 eV, confirming that these descriptors reflect the dominant factors. We also show that a more compact model using only four composite features still captures 86% of the variance and remains very robust in cross-validation tests. This stability, combined with its simplicity, makes the reduced model particularly attractive for practical applications. To further assess its transferability, we apply the model to a previously unseen surface facet, Ag(100), and to an entirely new host metal, Au(100). The DFT-based *d*-band widths for the same dopant in these two surfaces differ by no more than 0.02 eV, which is consistent with our design guidelines that identify the dopant–host distance, here reflected in the nearly identical lattice constants, as a primary parameter. The ML model predicts *d*-band widths for both newly added systems with an average deviation of only 0.06 eV from the DFT reference values, demonstrating its predictive power and applicability to a wide range of dilute alloy systems.

In addition to shaping the distinctive features of the dopants’ pDOSs, the spatial localization of their *d*-states can also lead to enhanced spin polarization. While high-spin states are a well-known characteristic of certain 3*d* TM dopants,^48,55^ we find that some 4*d* TM dopants can also favor high-spin configurations, depending on their local environment. In Figure 2, dopants for which a high-spin state is most stable are marked with empty symbols, and the *d*-band widths and centers for both spin channels are shown. Although the spin state affects the electronic structure, the overall periodic trends in *d*-band width remain intact.

The number of unpaired electrons (on-site magnetization, green diamonds) and the energetic stabilization of high-spin states relative to their corresponding low-spin states (pink circles) are shown in Figure 4. While no stable high-spin states are found for dopants embedded in bulk Cu, the number of dopants favoring high-spin configurations increases as confinement decreases, from two elements (Mo and Tc) in the most confining Cu(111) surface to five (Zr, Nb, Mo, Tc, and Ru) in the least confining Ag(211) surface. In addition to the increase in the number of elements exhibiting high-spin states, the number of unpaired electrons localized at a given dopant also grows with reduced confinement. For example, the magnetization of Mo increases from 0.0 μ_B_ in bulk Cu, to 1.2 μ_B_ on Cu(111), and up to 3.1 μ_B_ on Ag(211). This increase in unpaired electrons is accompanied by a substantial energetic stabilization of the high-spin state. While Mo adopts a nonmagnetic ground state in bulk Cu, the high-spin and low-spin states are nearly degenerate on Cu(111), with the high-spin configuration being only 1 kJ mol^–1^ more stable, well within the uncertainty of DFT. On Ag(211), by contrast, the high-spin state is favored by as much as 64 kJ mol^–1^, indicating a strong preference for the high-spin configuration.

**Figure 4:**
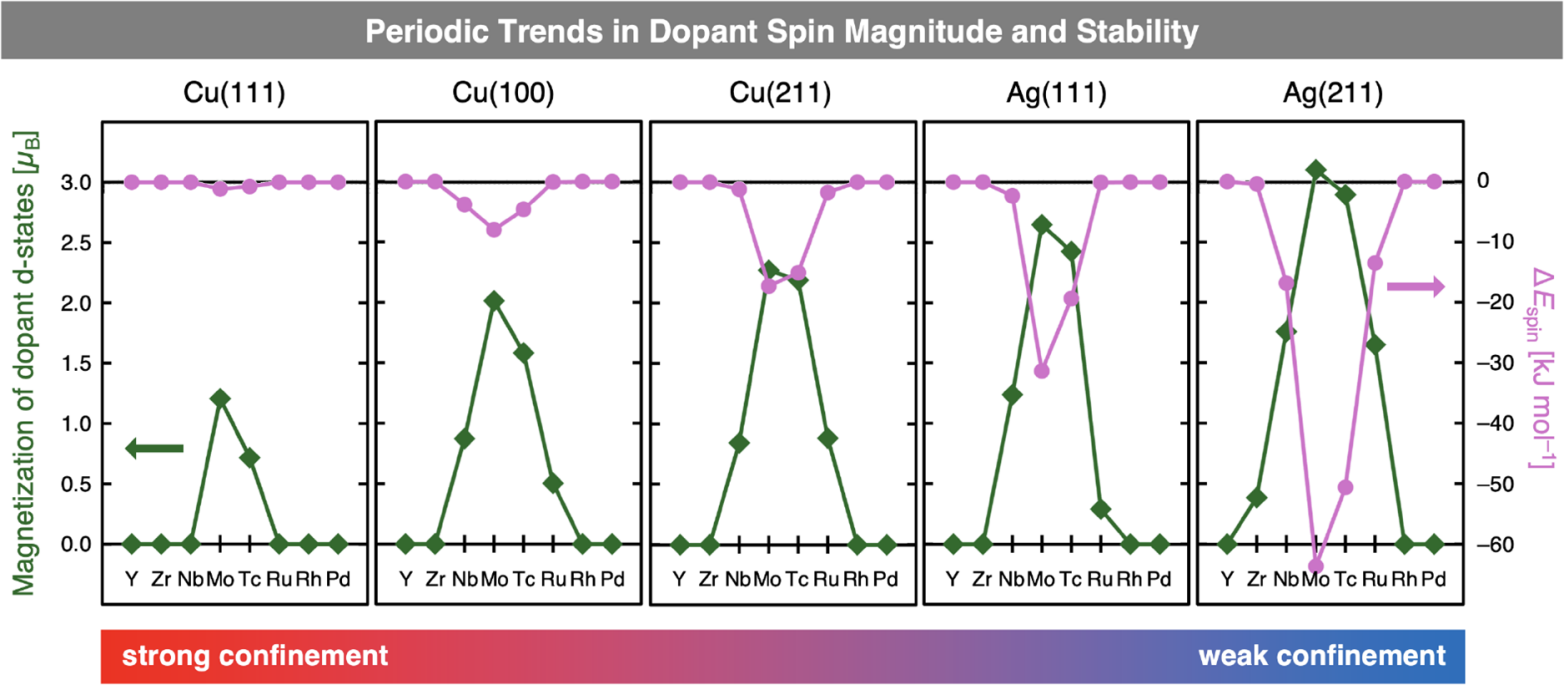
Periodic trends in the magnitude (green diamonds, left axis) and energetic stabilization (pink circles, right axis) of spin polarization for 4*d* TM dopants embedded in host metal surfaces. Central 4*d* TM elements exhibit spin polarization that becomes more pronounced and more energetically stabilized with decreasing dopant confinement. Connecting lines are for illustrative purposes only.

The dependence of magnetization on the individual effects of dopant–host distance and local symmetry is further examined in Section S6 in the Supporting Information, using Mo embedded in Cu surfaces with progressively expanded lattice constants, up to that of Ag. We find that both the magnitude of Mo’s magnetization and the energetic stabilization of its high-spin state increase with dopant–host distance, eventually reaching the values observed in Ag surfaces. This reinforces the conclusion that reduced dopant–host interaction enhances spin polarization. Consistent with the broader trends, local symmetry also plays a role: surface facets with fewer neighboring atoms further stabilize and amplify high-spin states. However, this facet-dependent effect diminishes as the lattice constant increases, indicating that the influence of the local symmetry weakens as the dopant becomes more spatially decoupled from the host.

High-spin states are not only characterized by localized unpaired electrons but also by spin-dependent splitting of the dopant pDOSs, as illustrated in Figures S1–S10 in the Supporting Information. Depending on the extent of this splitting: (i) for very small splitting, the total dopant pDOS can remain nearly unchanged (e.g., Zr in Ag(211)) compared to the low-spin configuration; (ii) for moderate splitting, the pDOS may appear broadened (e.g., Mo in Cu(100)); and (iii) for strong splitting, two distinct peaks may appear in the total dopant pDOS (e.g., Mo in Ag(211)). Thus, tuning dopant confinement also provides a pathway to modulate the spin state of active sites, which in turn can influence the catalytic properties of SAAs.^56^

To validate that the observed trends in *d*-band width and spin are not artifacts of the specific exchange–correlation functional used, we perform additional calculations for Zr, Mo, and Rh embedded in Cu(111) using two alternative generalized gradient approximation (GGA) functionals, RPBE^57^ and PBEsol,^58,59^ as well as the meta-GGA functional r^2^SCAN.^60^ These functionals are chosen for their distinct behavior.^61^ As detailed in Section S8 in the Supporting Information, we find that for nonmagnetic dopants such as Zr and Rh, the calculated *d*-band widths are not sensitive to the choice of functional. In contrast, magnetic properties, i.e., the magnitude of magnetization and the energetic stabilization of the high-spin state, do show some functional dependence. Specifically, r^2^SCAN predicts a more pronounced and more stabilized spin polarization for Mo compared to the GGA functionals. This can be attributed to the reduced self-interaction error in meta-GGAs, which can enhance the localization of states. Importantly, however, the *d*-band width, which is the central quantity of interest in this study, remains largely unaffected by the functional. The observed variations are significantly smaller than those induced by periodic trends or confinement effects, providing evidence for the robustness of our conclusions. Still, for future studies of SAAs where magnetic properties are expected to play a central role, particularly spin polarization in 3*d* TMs, we recommend the use of meta-GGA functionals such as r^2^SCAN.

Building on the trends and design strategies introduced above, we conclude by illustrating how variations in the *d*-band width of dopants at active sites can influence catalytic performance. For this purpose, we revisit literature data on the hydrogenation of crotonaldehyde,^29^ a reaction that can proceed via two competing pathways, with selectivity influenced by the choice of dopant in the SAA. As discussed in detail in Section S9 in the Supporting Information, we utilize reported adsorption energies^29^ and assume a BEP-like relation at the dopant sites to estimate product distributions. Our analysis reveals that selectivity is not only strongly influenced by the identity of the dopant element, but also by variations in the dopant’s *d*-band width depending on the local environment. For example, Rh, whose *d*-band width is rather insensitive to the host surface, shows only a small change in the predicted product ratio (a factor of 1.6) when moving from RhCu(111) to RhAg(111). In contrast, as discussed earlier, Zr experiences a substantial change in *d*-band width, leading to a dramatic shift in predicted product ratios by a factor of 261. Notably, these changes are not due to changing the dopant element itself, but rather due to modifying its electronic structure through the surrounding host. This example underscores the dopant *d*-band width as a central parameter for understanding, predicting, and ultimately guiding the rational design of catalysts with enhanced performance.

## Conclusions

Free-atom-like *d*-states have long been considered desirable for catalysis in single-atom catalysts. We explore the origins of periodic and confinement trends and translate them into practical design guidelines for tuning dopant properties. We further introduce an explicit distinction between the energetic distribution of *d*-states, as captured by the density of states, and the spatial distribution of electron density, which reflects the orbital character of the dopant. These two aspects are often simultaneously implied but not treated as separate components.

Specifically, we provide a unifying and quantitative perspective on the free-atom-like character of dopant *d*-states, synthesizing disparate aspects from the literature to create a cohesive understanding of the electronic structure of SAAs. We illustrate that the free-atom-like character of dopant *d*-states is not a general phenomenon in SAAs. Rather, this behavior is specific to late TM dopants, whereas early TMs exhibit significantly broader *d*-bands, often approaching the width of the host metal *d*-band. Consistent with prior work, we find that the dopant *d*-band width is strongly influenced by the local environment, and we show how it can be modified by varying the surface facet and host metal identity: more confined environments lead to broader dopant *d*-bands.

The electron distributions of the *d*-states for both early and late TMs resemble those of atomic orbitals, reflecting electronic decoupling from the host due to the energetic misalignment and avoided mixing between dopant and host states. Even for late TMs, whose *d*-states are closer in energy to the host *d*-band, state mixing remains weak. We attribute this, at least in part, to the energetic penalty of filling antibonding states, which would be formed through state-mixing, when both dopant and host *d*-bands are fully or nearly filled. Free-atom-like behavior, characterized by narrow pDOSs, requires both electronic decoupling and minimal spatial overlap between dopant *d*-states and the host environment. Late TMs satisfy this due to their smaller size and more compact *d*-states compared to early TMs.

In addition, confinement influences the dopant spin state: reduced confinement enhances spin polarization and stabilizes high-spin configurations. Together, these insights reveal how the dopant environment governs the electronic structure in SAAs, enabling new strategies for SAA design.^62,63^ To predict these changes and facilitate the use of the design principles, we provide a machine learning model that relies solely on simple structural and electronic descriptors without requiring any quantum chemical calculations. That these descriptors alone explain 92% of the variance in *d*-band widths further corroborates our understanding of its physical origins. The tunability explored here could enable the development of more selective and versatile active sites, particularly for reactions where early transition metals are desirable. We also demonstrate the direct impact of *d*-band width variation on product distribution for two competing reaction pathways in a model reaction. Our findings show that significant changes in the *d*-band width of the same dopant element across different host surfaces can lead to selectivity shifts of more than 2 orders of magnitude, whereas dopants whose *d*-band widths remain largely unchanged exhibit far smaller variations. Ultimately, this work helps to expands the design space for SAAs by providing guidance on how to achieve dopants with free-atom-like character, including elements typically exhibiting very broad *d*-bands such as early transition metals.

## Supplementary Material





## Notes

Computational details, densities of states, molecular orbital perspective on dopant–host state mixing, and quantification of dopant confinement (PDF)
Optimized structures, example input files, and analysis code (ZIP)
