# Supplementary material for: When Are Dopant d‑States Free-Atom-Like? Periodic Trends and Confinement Effects in Single-Atom Alloys
Source: J Am Chem Soc. 2025 Oct 3;147(41):37079–88. doi: 10.1021/jacs.5c07771 (PMC12532207; doi:10.1021/jacs.5c07771)
Supplement: Supplementary file 1 [file ja5c07771_si_001.pdf]

# Supporting Information: When Are Dopant d-States Free-Atom-Like? Periodic Trends and Confinement Effects in Single-Atom Alloys

Fabian Berger* and Angelos Michaelides*

*Yusuf Hamied Department of Chemistry, University of Cambridge, CB2 1EW Cambridge, UK*

E-mail: fb593@cam.ac.uk; am452@cam.ac.uk

# Contents

**S1 Computational Details** **S-3**

**S2 Densities of States** **S-4**

**S3 Molecular Orbital Perspective on Dopant and Host State Mixing** **S-20**

**S4 Periodic Trends in Dopant Protrusion** **S-22**

**S5 Quantification of Dopant Confinement** **S-25**

**S6 Separating Coordination, Spacing, and Finite-Size Effects** **S-27**

**S7 Machine Learning Model for Predicting Dopant *d*-Band Widths** **S-32**

**S8 Functional Dependence of *d*-Band Width and Spin Polarization** **S-40**

**S9 Impact of Dopant *d*-Band Width Variation on Catalytic Selectivity** **S-43**

**References** **S-47**

# S1 Computational Details

The metal surfaces are modeled using a 3×3×6 supercell, truncated at the (100), (111), and (211) facets, with a vacuum height of 15 Å and two fixed bottom layers. Periodic density functional theory (DFT) calculations were performed with the optB86b-vdW functional,[S1] as implemented in the Vienna Ab initio Simulation Package (VASP), version 6.3.0.[S2–S5] A plane-wave basis set was employed for valence electrons, along with the projector augmented wave (PAW) method for core electrons. PAW potentials recommended by VASP and the Materials Project[S6] were used. In cases where discrepancies arose, the potential requiring more explicitly treated electrons was selected (Cu: Cu, Ag: Ag, Y: Y_sv, Zr: Zr_sv, Nb: Nb_sv, Mo: Mo_sv, Tc: Tc_pv, Ru: Ru_pv, Rh: Rh_pv, Pd: Pd). Spin-polarization, first-order Methfessel-Paxton smearing (SIGMA = 0.1), and dipole correction were applied.

For structure optimizations, an energy cutoff of 500 eV was used, and the Brillouin zone was sampled with a Monkhorst-Pack mesh of 5×5×1 **k**-points. Convergence thresholds were set to $10^{-7}$eV for electronic energies and 0.01 eV $Å^{-1}$ for ionic forces. Wavefunctions required for electronic structure analysis were obtained from single-point calculations on the optimized structures, using a denser Monkhorst-Pack **k**-point mesh of 7×7×1. Example inputs are provided as separate files.

Unit cell vectors were determined using the equation of state method,[S7] as implemented in the Atomic Simulation Environment (ASE). An energy cutoff of 800 eV was used to minimize the influence of volume changes on the basis set, and the Brillouin zone of the bulk, represented by a primitive unit cell containing one atom, was sampled using a Monkhorst-Pack mesh with 31×31×31 **k**-points.

The band structure analysis was performed using the LOBSTER (Local Orbital Basis Suite Towards Electronic-Structure Reconstruction) program.[S8,S9]

# S2 Densities of States

**Table S1:** Electronic structure descriptors for dopants located in a **bulk layer of Cu** (layer 3 of 6). All dopants are predicted to exhibit a low-spin (ls) configuration without spin polarization, as indicated by the dopant magnetization ($m_{\text{dop}}$), which reflects the on-site number of unpaired electrons. Reported quantities include: the center of the main dopant $d$-band feature ($c_{\text{dop-ls}}$); dopant $d$-band filling up to the Fermi level ($f_{\text{dop-ls}}$); and the width of the main dopant feature, measured as the full width at half maximum (FWHM, $w_{\text{dop-ls}}$). Also shown is the upper edge of the host Cu $d$-band ($e_{\text{host}}$). All energy values are given in eV.

| TM | $m_{\text{dop}}$ | $c_{\text{dop-ls}}$ | $f_{\text{dop-ls}}$ | $w_{\text{dop-ls}}$ | $e_{\text{host}}$ |
|---|---|---|---|---|---|
| Y | 0.00 | 1.83 | 2.68 | 2.56 | −1.55 |
| Zr | 0.00 | 1.38 | 3.31 | 2.41 | −1.60 |
| Nb | 0.00 | 0.86 | 4.57 | 2.10 | −1.55 |
| Mo | 0.00 | 0.56 | 5.62 | 0.64 | −1.55 |
| Tc | 0.00 | 0.40 | 6.45 | 0.69 | −1.50 |
| Ru | 0.00 | 0.08 | 7.74 | 0.79 | −1.45 |
| Rh | 0.00 | −0.37 | 8.71 | 0.54 | −1.55 |
| Pd | 0.00 | −1.23 | 9.50 | 0.29 | −1.55 |
| Ag | 0.00 | −5.86 | 9.83 | 0.36 | - |
| Cd | 0.00 | −9.21 | 9.95 | 0.23 | - |

**Table S2:** Electronic structure descriptors for dopants located in the **Cu(111)** surface. For all dopants, the predicted low-spin (ls) configuration without spin polarization is shown. For cases where a high-spin configuration is more stable than the spin unpolarized configuration, the energy difference ($\Delta E$) as well as the spin-resolved alpha ($\alpha$) and beta ($\beta$) components are also reported. Reported quantities include: the dopant magnetization of the most stable spin state ($m_{\text{dop}}$), reflecting the on-site number of unpaired electrons; the center of the main dopant $d$-band feature ($c_{\text{dop-ls}}$, $c_{\text{dop-}\alpha}$, $c_{\text{dop-}\beta}$); the dopant $d$-band filling up to the Fermi level ($f_{\text{dop-ls}}$, $f_{\text{dop-}\alpha}$, $f_{\text{dop-}\beta}$); and the width of the main dopant $d$-band feature measured as the full width at half maximum (FWHM: $w_{\text{dop-ls}}$, $w_{\text{dop-}\alpha}$, $w_{\text{dop-}\beta}$). Also shown is the upper edge of the Cu host $d$-band ($e_{\text{host}}$). All energies are reported in eV.

| TM | $m_{\text{dop}}$ | $\Delta E$ | $c_{\text{dop-ls}}$ | $c_{\text{dop-}\alpha}$ | $c_{\text{dop-}\beta}$ | $f_{\text{dop-ls}}$ | $f_{\text{dop-}\alpha}$ | $f_{\text{dop-}\beta}$ | $w_{\text{dop-ls}}$ | $w_{\text{dop-}\alpha}$ | $w_{\text{dop-}\beta}$ | $e_{\text{host}}$ |
|---|---|---|---|---|---|---|---|---|---|---|---|---|
| Y | 0.00 | - | 1.55 | - | - | 1.87 | - | - | 2.25 | - | - | $-1.55$ |
| Zr | 0.00 | - | 1.07 | - | - | 3.01 | - | - | 2.37 | - | - | $-1.55$ |
| Nb | 0.00 | - | 0.69 | - | - | 4.38 | - | - | 1.30 | - | - | $-1.55$ |
| Mo | 1.21 | 0.01 | 0.47 | 0.18 | 0.76 | 5.47 | 3.49 | 1.97 | 1.24 | 0.75 | 1.22 | $-1.55$ |
| Tc | 0.72 | 0.01 | 0.32 | 0.05 | 0.55 | 6.40 | 3.84 | 2.52 | 0.74 | 0.69 | 1.20 | $-1.55$ |
| Ru | 0.00 | - | 0.04 | - | - | 7.67 | - | - | 0.57 | - | - | $-1.50$ |
| Rh | 0.00 | - | $-0.33$ | - | - | 8.69 | - | - | 0.45 | - | - | $-1.55$ |
| Pd | 0.00 | - | $-1.37$ | - | - | 9.51 | - | - | 0.29 | - | - | $-1.55$ |
| Ag | 0.00 | - | $-4.98$ | - | - | 9.85 | - | - | 0.74 | - | - | - |
| Cd | 0.00 | - | $-8.54$ | - | - | 9.97 | - | - | 0.22 | - | - | - |

**Table S3:** Electronic structure descriptors for dopants located in the **Cu(100)** surface. For all dopants, the predicted low-spin (ls) configuration without spin polarization is shown. For cases where a high-spin configuration is more stable than the spin unpolarized configuration, the energy difference ($\Delta E$) as well as the spin-resolved alpha ($\alpha$) and beta ($\beta$) components are also reported. Reported quantities include: the dopant magnetization of the most stable spin state ($m_{\text{dop}}$), reflecting the on-site number of unpaired electrons; the center of the main dopant $d$-band feature ($c_{\text{dop-ls}}$, $c_{\text{dop-}\alpha}$, $c_{\text{dop-}\beta}$); the dopant $d$-band filling up to the Fermi level ($f_{\text{dop-ls}}$, $f_{\text{dop-}\alpha}$, $f_{\text{dop-}\beta}$); and the width of the main dopant $d$-band feature measured as the full width at half maximum (FWHM: $w_{\text{dop-ls}}$, $w_{\text{dop-}\alpha}$, $w_{\text{dop-}\beta}$). Also shown is the upper edge of the Cu host $d$-band ($e_{\text{host}}$). All energies are reported in eV.

| TM | $m_{\text{dop}}$ | $\Delta E$ | $c_{\text{dop-ls}}$ | $c_{\text{dop-}\alpha}$ | $c_{\text{dop-}\beta}$ | $f_{\text{dop-ls}}$ | $f_{\text{dop-}\alpha}$ | $f_{\text{dop-}\beta}$ | $w_{\text{dop-ls}}$ | $w_{\text{dop-}\alpha}$ | $w_{\text{dop-}\beta}$ | $e_{\text{host}}$ |
|---|---|---|---|---|---|---|---|---|---|---|---|---|
| Y | 0.00 | - | 1.48 | - | - | 1.81 | - | - | 1.38 | - | - | −1.50 |
| Zr | 0.00 | - | 0.96 | - | - | 2.87 | - | - | 1.21 | - | - | −1.50 |
| Nb | 0.87 | 0.04 | 0.64 | 0.44 | 0.87 | 4.24 | 2.71 | 1.56 | 1.12 | 0.65 | 0.91 | −1.50 |
| Mo | 2.01 | 0.08 | 0.42 | −0.08 | 0.94 | 5.42 | 3.94 | 1.39 | 0.65 | 0.55 | 1.27 | −1.50 |
| Tc | 1.58 | 0.05 | 0.28 | −0.18 | 0.61 | 6.36 | 4.14 | 2.17 | 0.61 | 0.46 | 0.88 | −1.50 |
| Ru | 0.50 | 0.00 | 0.01 | −0.02 | 0.03 | 7.68 | 3.91 | 3.77 | 0.56 | 0.54 | 0.56 | −1.50 |
| Rh | 0.00 | - | −0.31 | - | - | 8.70 | - | - | 0.41 | - | - | −1.50 |
| Pd | 0.00 | - | −1.07 | - | - | 9.54 | - | - | 0.33 | - | - | −1.50 |
| Ag | 0.00 | - | −4.68 | - | - | 9.87 | - | - | 0.71 | - | - | - |
| Cd | 0.00 | - | −8.58 | - | - | 9.97 | - | - | 0.23 | - | - | - |

**Table S4:** Electronic structure descriptors for dopants located at the step edge of the **Cu(211)** surface. For all dopants, the predicted low-spin (ls) configuration without spin polarization is shown. For cases where a high-spin configuration is more stable than the spin unpolarized configuration, the energy difference ($\Delta E$) as well as the spin-resolved alpha ($\alpha$) and beta ($\beta$) components are also reported. Reported quantities include: the dopant magnetization of the most stable spin state ($m_{\text{dop}}$), reflecting the on-site number of unpaired electrons; the center of the main dopant $d$-band feature ($c_{\text{dop-ls}}$, $c_{\text{dop-}\alpha}$, $c_{\text{dop-}\beta}$); the dopant $d$-band filling up to the Fermi level ($f_{\text{dop-ls}}$, $f_{\text{dop-}\alpha}$, $f_{\text{dop-}\beta}$); and the width of the main dopant $d$-band feature measured as the full width at half maximum (FWHM: $w_{\text{dop-ls}}$, $w_{\text{dop-}\alpha}$, $w_{\text{dop-}\beta}$). Also shown is the upper edge of the Cu host $d$-band ($e_{\text{host}}$). All energies are reported in eV.

| TM | $m_{\text{dop}}$ | $\Delta E$ | $c_{\text{dop-ls}}$ | $c_{\text{dop-}\alpha}$ | $c_{\text{dop-}\beta}$ | $f_{\text{dop-ls}}$ | $f_{\text{dop-}\alpha}$ | $f_{\text{dop-}\beta}$ | $w_{\text{dop-ls}}$ | $w_{\text{dop-}\alpha}$ | $w_{\text{dop-}\beta}$ | $e_{\text{host}}$ |
|---|---|---|---|---|---|---|---|---|---|---|---|---|
| Y | 0.00 | - | 1.45 | - | - | 1.72 | - | - | 1.61 | - | - | −1.50 |
| Zr | 0.00 | - | 0.92 | - | - | 2.84 | - | - | 1.61 | - | - | −1.55 |
| Nb | 0.84 | 0.01 | 0.57 | 0.39 | 0.85 | 4.24 | 2.65 | 1.54 | 1.13 | 1.06 | 1.29 | −1.55 |
| Mo | 2.27 | 0.18 | 0.39 | −0.15 | 0.98 | 5.34 | 4.01 | 1.30 | 0.80 | 0.69 | 1.55 | −1.50 |
| Tc | 2.19 | 0.16 | 0.27 | −0.36 | 0.63 | 6.32 | 4.32 | 1.92 | 0.72 | 0.54 | 1.08 | −1.50 |
| Ru | 0.00 | - | 0.00 | - | - | 7.68 | - | - | 0.60 | - | - | −1.50 |
| Rh | 0.00 | - | −0.32 | - | - | 8.68 | - | - | 0.53 | - | - | −1.50 |
| Pd | 0.00 | - | −1.17 | - | - | 9.52 | - | - | 0.34 | - | - | −1.50 |
| Ag | 0.00 | - | −4.52 | - | - | 9.88 | - | - | 0.78 | - | - | - |
| Cd | 0.00 | - | −8.53 | - | - | 9.97 | - | - | 0.24 | - | - | - |

**Table S5:** Electronic structure descriptors for dopants located in the **Ag(111)** surface. For all dopants, the predicted low-spin (ls) configuration without spin polarization is shown. For cases where a high-spin configuration is more stable than the spin unpolarized configuration, the energy difference ($\Delta E$) as well as the spin-resolved alpha ($\alpha$) and beta ($\beta$) components are also reported. Reported quantities include: the dopant magnetization of the most stable spin state ($m_{\text{dop}}$), reflecting the on-site number of unpaired electrons; the center of the main dopant $d$-band feature ($c_{\text{dop-ls}}$, $c_{\text{dop-}\alpha}$, $c_{\text{dop-}\beta}$); the dopant $d$-band filling up to the Fermi level ($f_{\text{dop-ls}}$, $f_{\text{dop-}\alpha}$, $f_{\text{dop-}\beta}$); and the width of the main dopant $d$-band feature measured as the full width at half maximum (FWHM: $w_{\text{dop-ls}}$, $w_{\text{dop-}\alpha}$, $w_{\text{dop-}\beta}$). Also shown is the upper edge of the Cu host $d$-band ($e_{\text{host}}$). All energies are reported in eV.

| TM | $m_{\text{dop}}$ | $\Delta E$ | $c_{\text{dop-ls}}$ | $c_{\text{dop-}\alpha}$ | $c_{\text{dop-}\beta}$ | $f_{\text{dop-ls}}$ | $f_{\text{dop-}\alpha}$ | $f_{\text{dop-}\beta}$ | $w_{\text{dop-ls}}$ | $w_{\text{dop-}\alpha}$ | $w_{\text{dop-}\beta}$ | $e_{\text{host}}$ |
|---|---|---|---|---|---|---|---|---|---|---|---|---|
| Y | 0.00 | - | 1.41 | - | - | 1.69 | - | - | 1.84 | - | - | −2.91 |
| Zr | 0.00 | - | 0.87 | - | - | 2.76 | - | - | 1.12 | - | - | −2.91 |
| Nb | 1.24 | 0.02 | 0.44 | 0.11 | 0.98 | 4.14 | 2.99 | 1.09 | 1.10 | 0.92 | 0.68 | −2.91 |
| Mo | 2.65 | 0.32 | 0.23 | −0.54 | 1.09 | 5.24 | 4.29 | 0.87 | 0.85 | 0.50 | 0.66 | −2.91 |
| Tc | 2.42 | 0.20 | 0.12 | −0.85 | 0.54 | 6.16 | 4.48 | 1.62 | 0.74 | 0.44 | 1.07 | −2.86 |
| Ru | 0.29 | 0.00 | −0.12 | −0.43 | 0.05 | 7.56 | 4.26 | 3.22 | 0.59 | 0.41 | 0.67 | −2.86 |
| Rh | 0.00 | - | −0.48 | - | - | 8.65 | - | - | 0.39 | - | - | −2.86 |
| Pd | 0.00 | - | −1.39 | - | - | 9.55 | - | - | 0.32 | - | - | −2.91 |
| Ag | 0.00 | - | - | - | - | - | - | - | - | - | - | - |
| Cd | 0.00 | - | −8.62 | - | - | 9.98 | - | - | 0.22 | - | - | - |

**Table S6:** Electronic structure descriptors for dopants located at the step edge of the **Ag(211)** surface. For all dopants, the predicted low-spin (ls) configuration without spin polarization is shown. For cases where a high-spin configuration is more stable than the spin unpolarized configuration, the energy difference ($\Delta E$) as well as the spin-resolved alpha ($\alpha$) and beta ($\beta$) components are also reported. Reported quantities include: the dopant magnetization of the most stable spin state ($m_{\text{dop}}$), reflecting the on-site number of unpaired electrons; the center of the main dopant $d$-band feature ($c_{\text{dop-ls}}$, $c_{\text{dop-}\alpha}$, $c_{\text{dop-}\beta}$); the dopant $d$-band filling up to the Fermi level ($f_{\text{dop-ls}}$, $f_{\text{dop-}\alpha}$, $f_{\text{dop-}\beta}$); and the width of the main dopant $d$-band feature measured as the full width at half maximum (FWHM: $w_{\text{dop-ls}}$, $w_{\text{dop-}\alpha}$, $w_{\text{dop-}\beta}$). Also shown is the upper edge of the Cu host $d$-band ($e_{\text{host}}$). All energies are reported in eV.

| TM | $m_{\text{dop}}$ | $\Delta E$ | $c_{\text{dop-ls}}$ | $c_{\text{dop-}\alpha}$ | $c_{\text{dop-}\beta}$ | $f_{\text{dop-ls}}$ | $f_{\text{dop-}\alpha}$ | $f_{\text{dop-}\beta}$ | $w_{\text{dop-ls}}$ | $w_{\text{dop-}\alpha}$ | $w_{\text{dop-}\beta}$ | $e_{\text{host}}$ |
|---|---|---|---|---|---|---|---|---|---|---|---|---|
| Y | 0.00 | - | 1.21 | - | - | 1.56 | - | - | 1.21 | - | - | −2.86 |
| Zr | 0.38 | 0.00 | 0.70 | 0.64 | 0.69 | 2.71 | 1.40 | 1.30 | 1.02 | 1.02 | 1.02 | −2.86 |
| Nb | 1.76 | 0.17 | 0.38 | −0.02 | 1.19 | 4.06 | 3.30 | 0.74 | 1.14 | 0.92 | 1.20 | −2.86 |
| Mo | 3.10 | 0.66 | 0.19 | −0.67 | 1.22 | 5.21 | 4.45 | 0.64 | 0.90 | 0.52 | 1.21 | −2.86 |
| Tc | 2.89 | 0.52 | 0.09 | −1.13 | 0.56 | 6.14 | 4.66 | 1.36 | 0.75 | 0.37 | 0.91 | −2.86 |
| Ru | 1.65 | 0.14 | −0.11 | −0.82 | 0.10 | 7.53 | 4.60 | 2.83 | 0.58 | 0.54 | 0.90 | −2.86 |
| Rh | 0.00 | - | −0.43 | - | - | 8.66 | - | - | 0.49 | - | - | −2.86 |
| Pd | 0.00 | - | −1.19 | - | - | 9.56 | - | - | 0.36 | - | - | −2.86 |
| Ag | 0.00 | - | - | - | - | - | - | - | - | - | - | - |
| Cd | 0.00 | - | −8.58 | - | - | 9.98 | - | - | 0.20 | - | - | - |

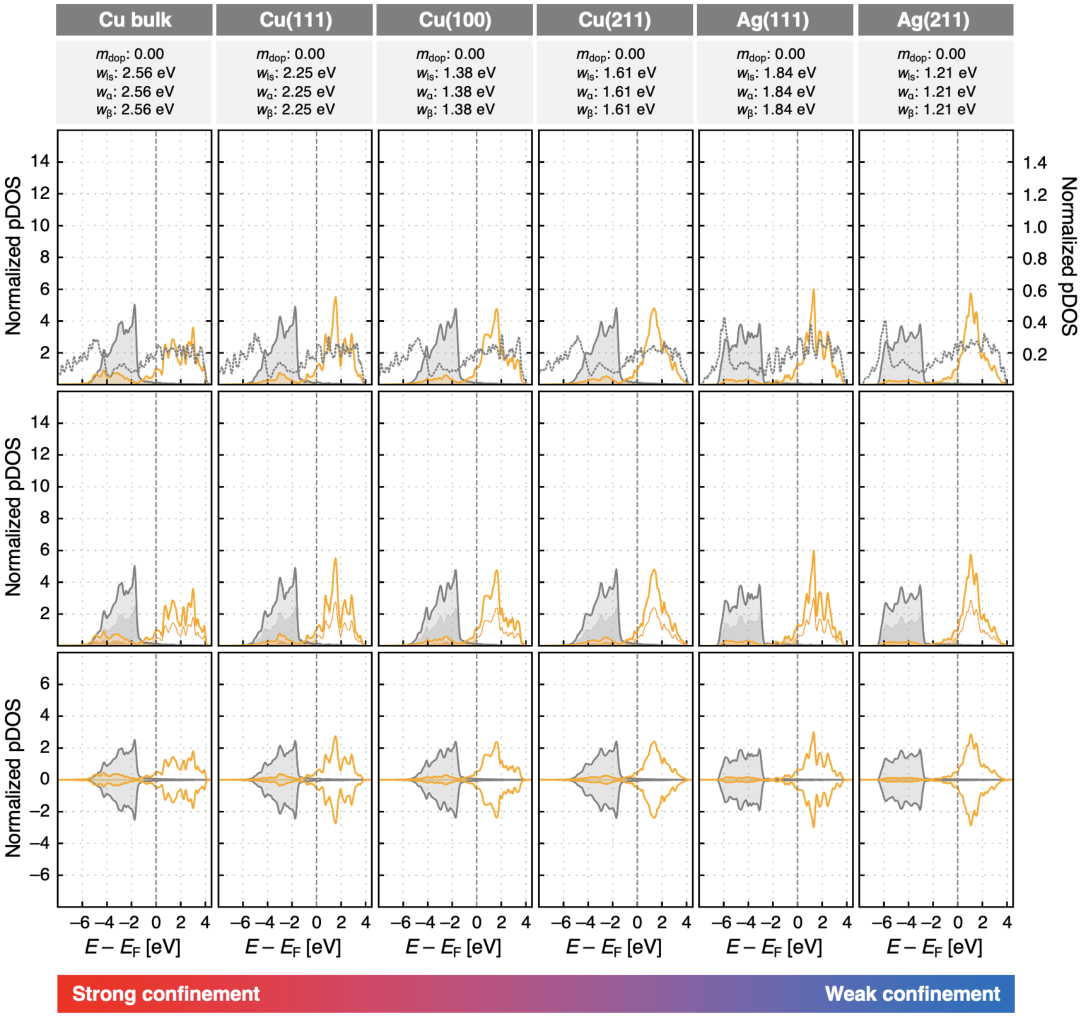


**Figure S1:** Projected densities of states (pDOS) for the most stable spin configuration ($m_{dop}$) of the **dopant Y** (orange) and the Cu and Ag host surfaces (gray), normalized per atom. The width of the main dopant $d$-band feature, measured as the full width at half maximum in the spin-unpolarized ($w_{\rm ls}$), alpha ($w_\alpha$), and beta ($w_\beta$) spin channels, tends to decrease with reduced dopant confinement. Shaded areas indicate $d$-band filling up to the Fermi level ($E_{\rm F}$, gray dashed vertical line). **Top panels**: Solid lines represent the pDOS of dopant and host $d$-states; dotted lines show host $s$-states. The largely unaltered shape of the host $s$-state pDOS indicates minimal mixing with dopant $d$-states. **Mid panels**: Cumulative pDOS of dopant and host $d$-states including both alpha and beta spin channels. Beta contributions (light gray, light orange) are stacked atop alpha contributions (dark gray, dark orange), separated by a line. **Bottom panels**: Spin-resolved pDOS of dopant and host $d$-states, with alpha (positive) and beta (negative) spin contributions shown with opposite signs.

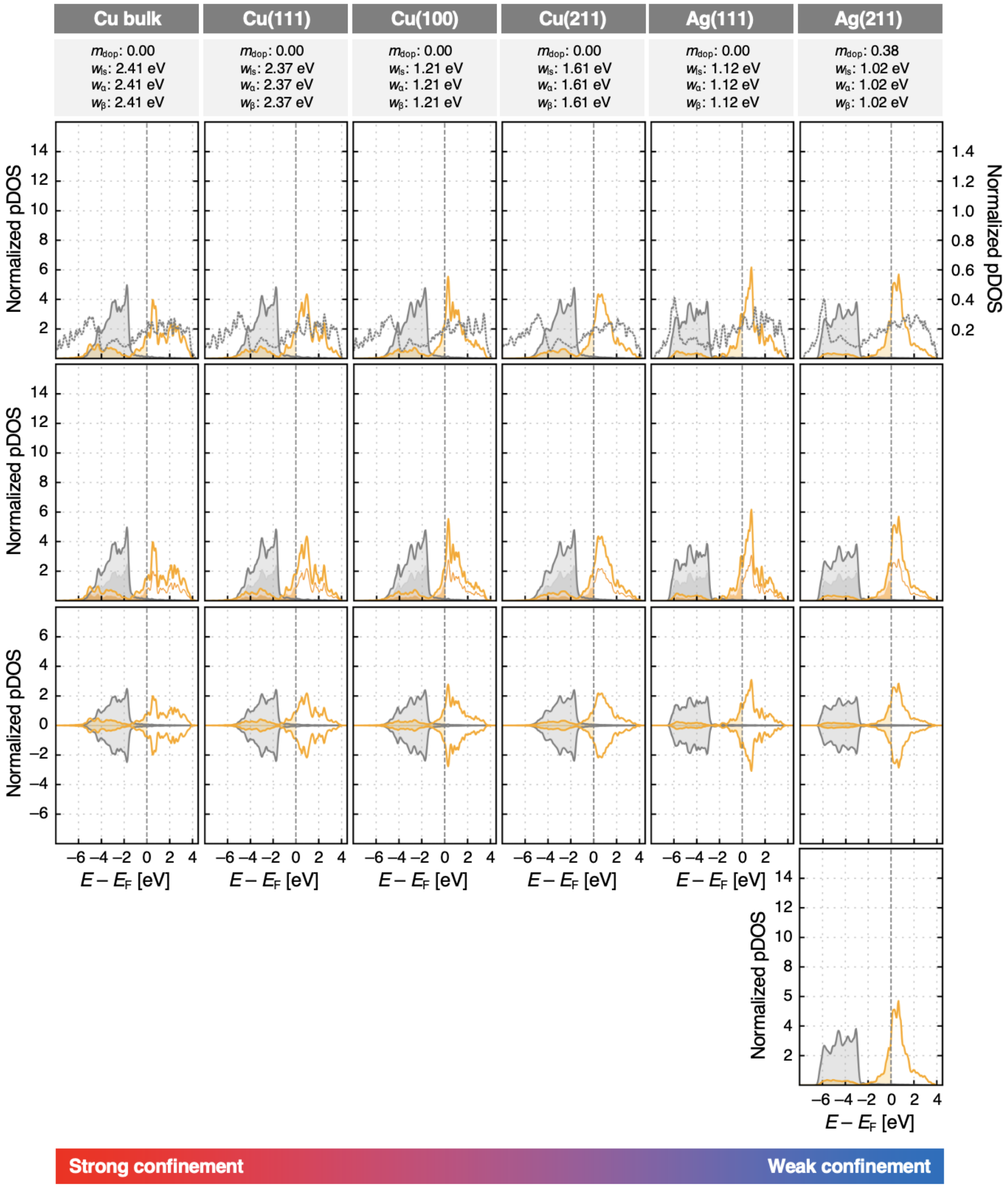


**Figure S2:** Projected densities of states (pDOS) for the most stable spin configuration ($m_{dop}$) of the **dopant Zr** (orange) and the Cu and Ag host surfaces (gray), normalized per atom. The width of the main dopant $d$-band feature, measured as the full width at half maximum in the spin-unpolarized ($w_{\text{ls}}$), alpha ($w_\alpha$), and beta ($w_\beta$) spin channels, tends to decrease with reduced dopant confinement. Shaded areas indicate $d$-band filling up to the Fermi level ($E_{\text{F}}$, gray dashed vertical line). **Top panels**: Solid lines represent the pDOS of dopant and host $d$-states; dotted lines show host $s$-states. The largely unaltered shape of the host $s$-state pDOS indicates minimal mixing with dopant $d$-states. **Upper middle panels**: Cumulative pDOS of dopant and host $d$-states including both alpha and beta spin channels. Beta contributions (light gray, light orange) are stacked atop alpha contributions (dark gray, dark orange), separated by a line. **Lower middle panels**: Spin-resolved pDOS of dopant and host $d$-states, with alpha (positive) and beta (negative) spin contributions shown with opposite signs. **Bottom panels**: pDOS of spin-unpolarized dopant and host $d$-states, shown for comparison when the system adopts a spin-polarized ground state.

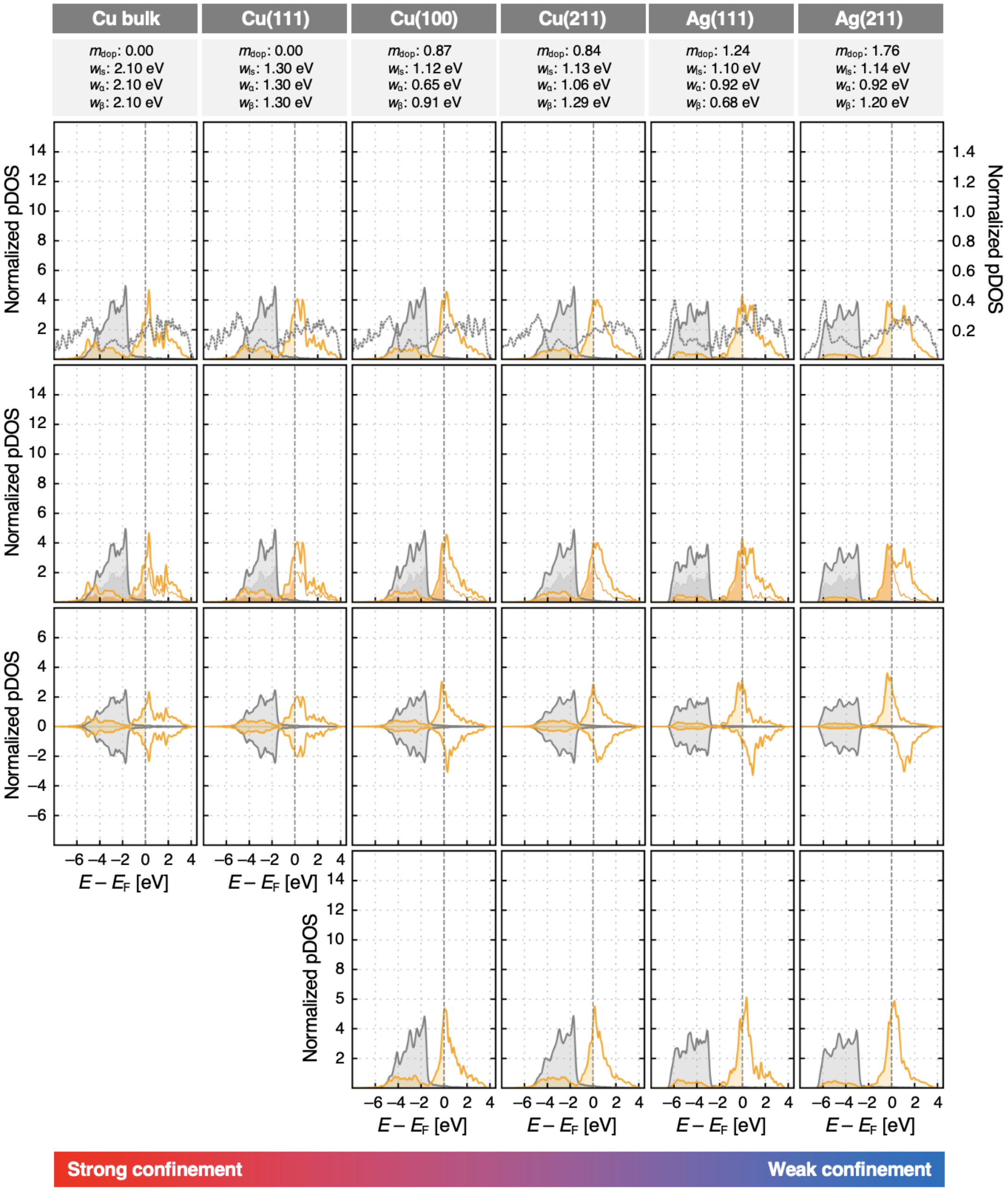


**Figure S3:** Projected densities of states (pDOS) for the most stable spin configuration ($m_{dop}$) of the **dopant Nb** (orange) and the Cu and Ag host surfaces (gray), normalized per atom. The width of the main dopant $d$-band feature, measured as the full width at half maximum in the spin-unpolarized ($w_{\rm ls}$), alpha ($w_\alpha$), and beta ($w_\beta$) spin channels, tends to decrease with reduced dopant confinement. Shaded areas indicate $d$-band filling up to the Fermi level ($E_{\rm F}$, gray dashed vertical line). **Top panels**: Solid lines represent the pDOS of dopant and host $d$-states; dotted lines show host $s$-states. The largely unaltered shape of the host $s$-state pDOS indicates minimal mixing with dopant $d$-states. **Upper middle panels**: Cumulative pDOS of dopant and host $d$-states including both alpha and beta spin channels. Beta contributions (light gray, light orange) are stacked atop alpha contributions (dark gray, dark orange), separated by a line. **Lower middle panels**: Spin-resolved pDOS of dopant and host $d$-states, with alpha (positive) and beta (negative) spin contributions shown with opposite signs. **Bottom panels**: pDOS of spin-unpolarized dopant and host $d$-states, shown for comparison when the system adopts a spin-polarized ground state.

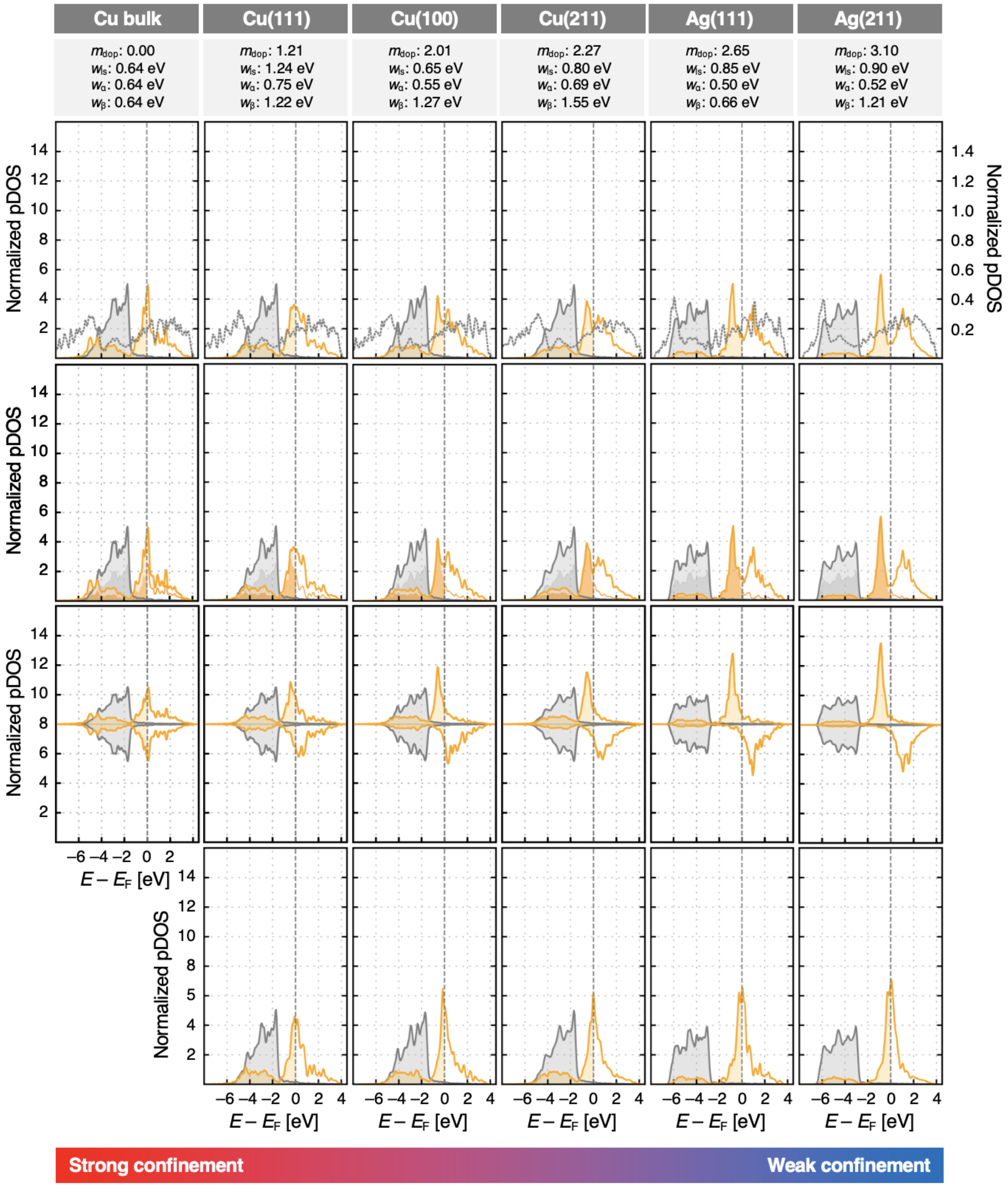


**Figure S4:** Projected densities of states (pDOS) for the most stable spin configuration ($m_{dop}$) of the **dopant Mo** (orange) and the Cu and Ag host surfaces (gray), normalized per atom. The width of the main dopant $d$-band feature, measured as the full width at half maximum in the spin-unpolarized ($w_{\mathrm{ls}}$), alpha ($w_\alpha$), and beta ($w_\beta$) spin channels, tends to decrease with reduced dopant confinement. Shaded areas indicate $d$-band filling up to the Fermi level ($E_{\mathrm{F}}$, gray dashed vertical line). **Top panels**: Solid lines represent the pDOS of dopant and host $d$-states; dotted lines show host $s$-states. The largely unaltered shape of the host $s$-state pDOS indicates minimal mixing with dopant $d$-states. **Upper middle panels**: Cumulative pDOS of dopant and host $d$-states including both alpha and beta spin channels. Beta contributions (light gray, light orange) are stacked atop alpha contributions (dark gray, dark orange), separated by a line. **Lower middle panels**: Spin-resolved pDOS of dopant and host $d$-states, with alpha (positive) and beta (negative) spin contributions shown with opposite signs. **Bottom panels**: pDOS of spin-unpolarized dopant and host $d$-states, shown for comparison when the system adopts a spin-polarized ground state.

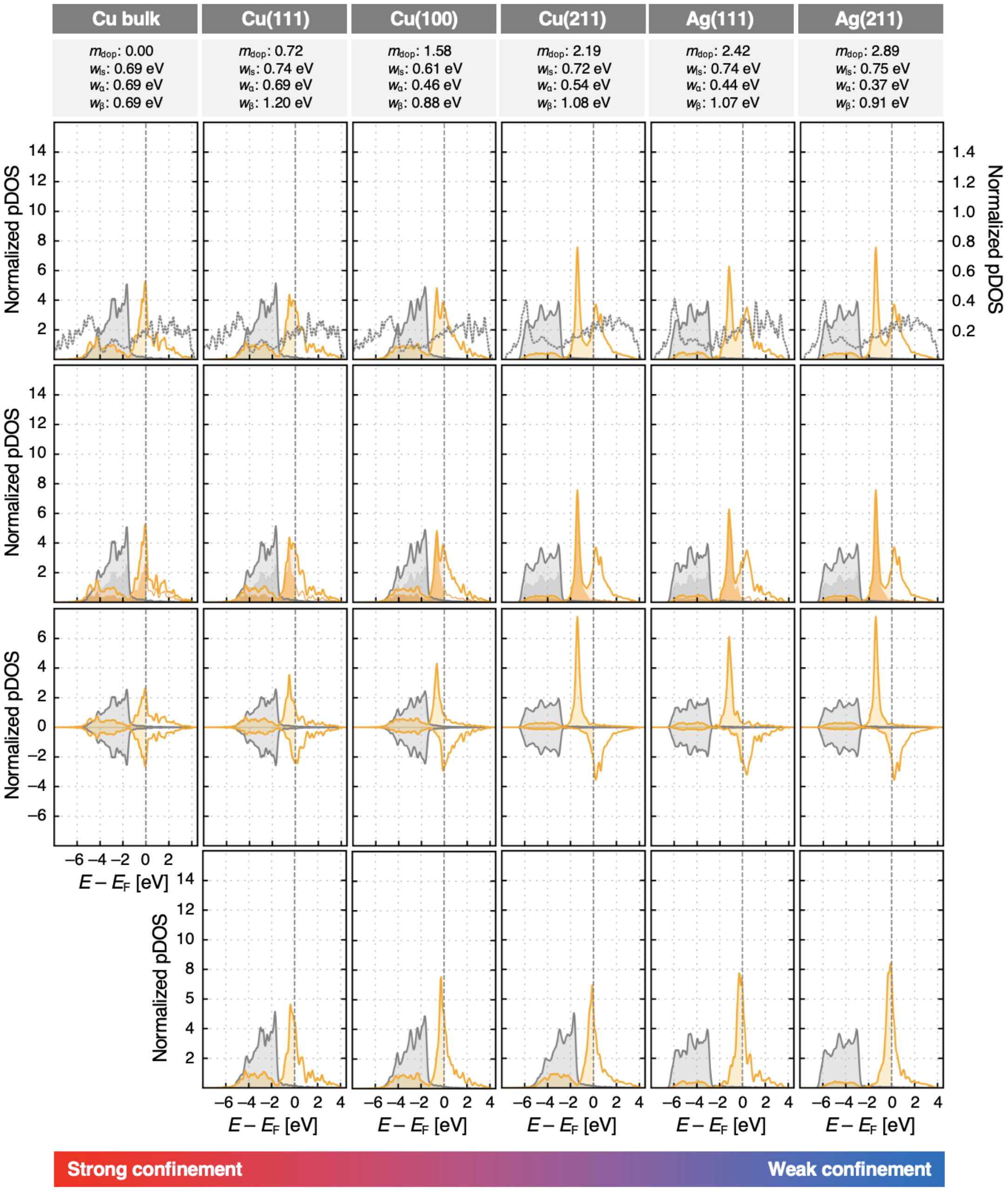


**Figure S5:** Projected densities of states (pDOS) for the most stable spin configuration ($m_{dop}$) of the **dopant Tc** (orange) and the Cu and Ag host surfaces (gray), normalized per atom. The width of the main dopant $d$-band feature, measured as the full width at half maximum in the spin-unpolarized ($w_{\text{ls}}$), alpha ($w_\alpha$), and beta ($w_\beta$) spin channels, tends to decrease with reduced dopant confinement. Shaded areas indicate $d$-band filling up to the Fermi level ($E_\text{F}$, gray dashed vertical line). **Top panels**: Solid lines represent the pDOS of dopant and host $d$-states; dotted lines show host $s$-states. The largely unaltered shape of the host $s$-state pDOS indicates minimal mixing with dopant $d$-states. **Upper middle panels**: Cumulative pDOS of dopant and host $d$-states including both alpha and beta spin channels. Beta contributions (light gray, light orange) are stacked atop alpha contributions (dark gray, dark orange), separated by a line. **Lower middle panels**: Spin-resolved pDOS of dopant and host $d$-states, with alpha (positive) and beta (negative) spin contributions shown with opposite signs. **Bottom panels**: pDOS of spin-unpolarized dopant and host $d$-states, shown for comparison when the system adopts a spin-polarized ground state.

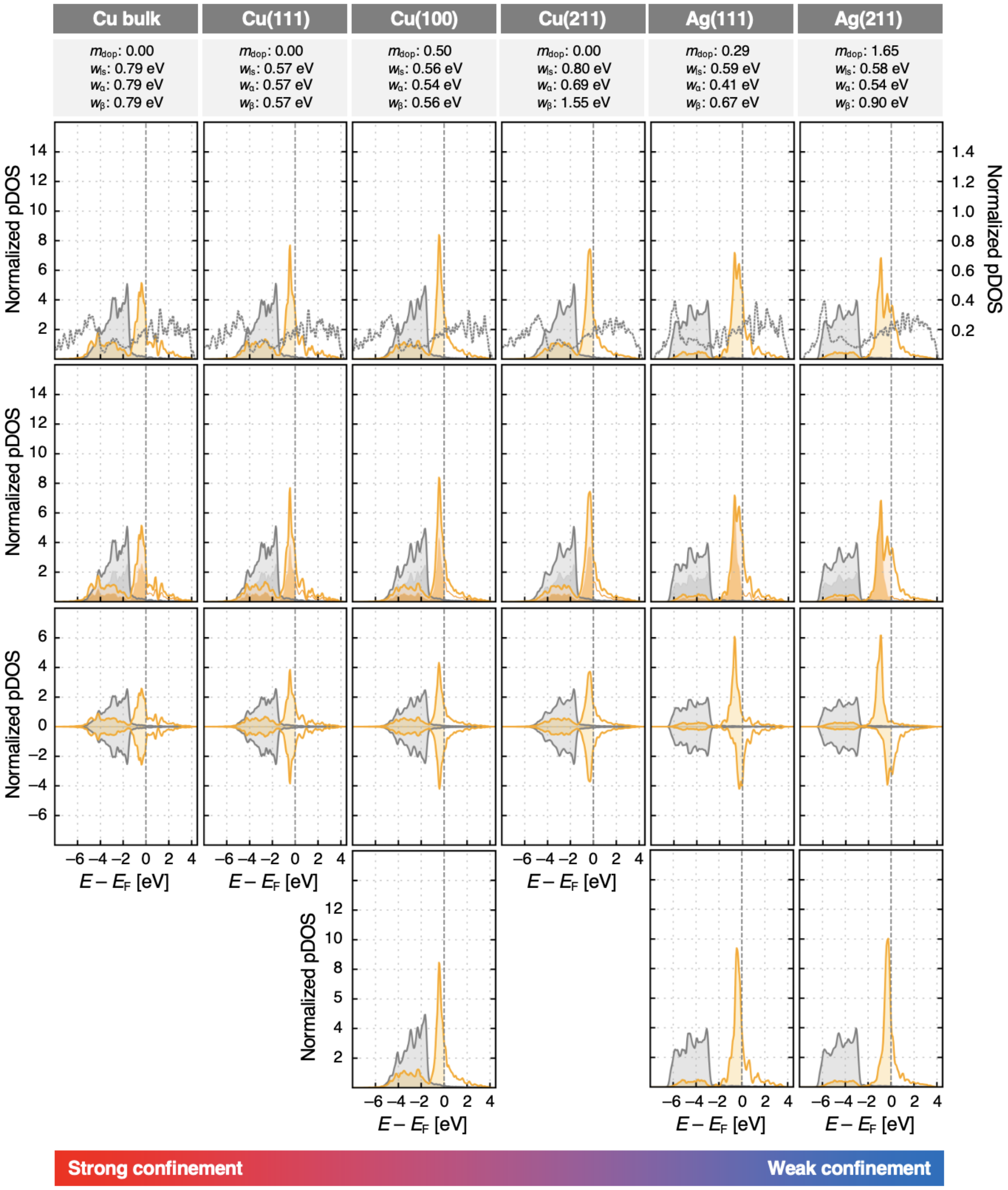


**Figure S6:** Projected densities of states (pDOS) for the most stable spin configuration ($m_{dop}$) of the **dopant Ru** (orange) and the Cu and Ag host surfaces (gray), normalized per atom. The width of the main dopant $d$-band feature, measured as the full width at half maximum in the spin-unpolarized ($w_{\mathrm{ls}}$), alpha ($w_\alpha$), and beta ($w_\beta$) spin channels, tends to decrease with reduced dopant confinement. Shaded areas indicate $d$-band filling up to the Fermi level ($E_{\mathrm{F}}$, gray dashed vertical line). **Top panels**: Solid lines represent the pDOS of dopant and host $d$-states; dotted lines show host $s$-states. The largely unaltered shape of the host $s$-state pDOS indicates minimal mixing with dopant $d$-states. **Upper middle panels**: Cumulative pDOS of dopant and host $d$-states including both alpha and beta spin channels. Beta contributions (light gray, light orange) are stacked atop alpha contributions (dark gray, dark orange), separated by a line. **Lower middle panels**: Spin-resolved pDOS of dopant and host $d$-states, with alpha (positive) and beta (negative) spin contributions shown with opposite signs. **Bottom panels**: pDOS of spin-unpolarized dopant and host $d$-states, shown for comparison when the system adopts a spin-polarized ground state.

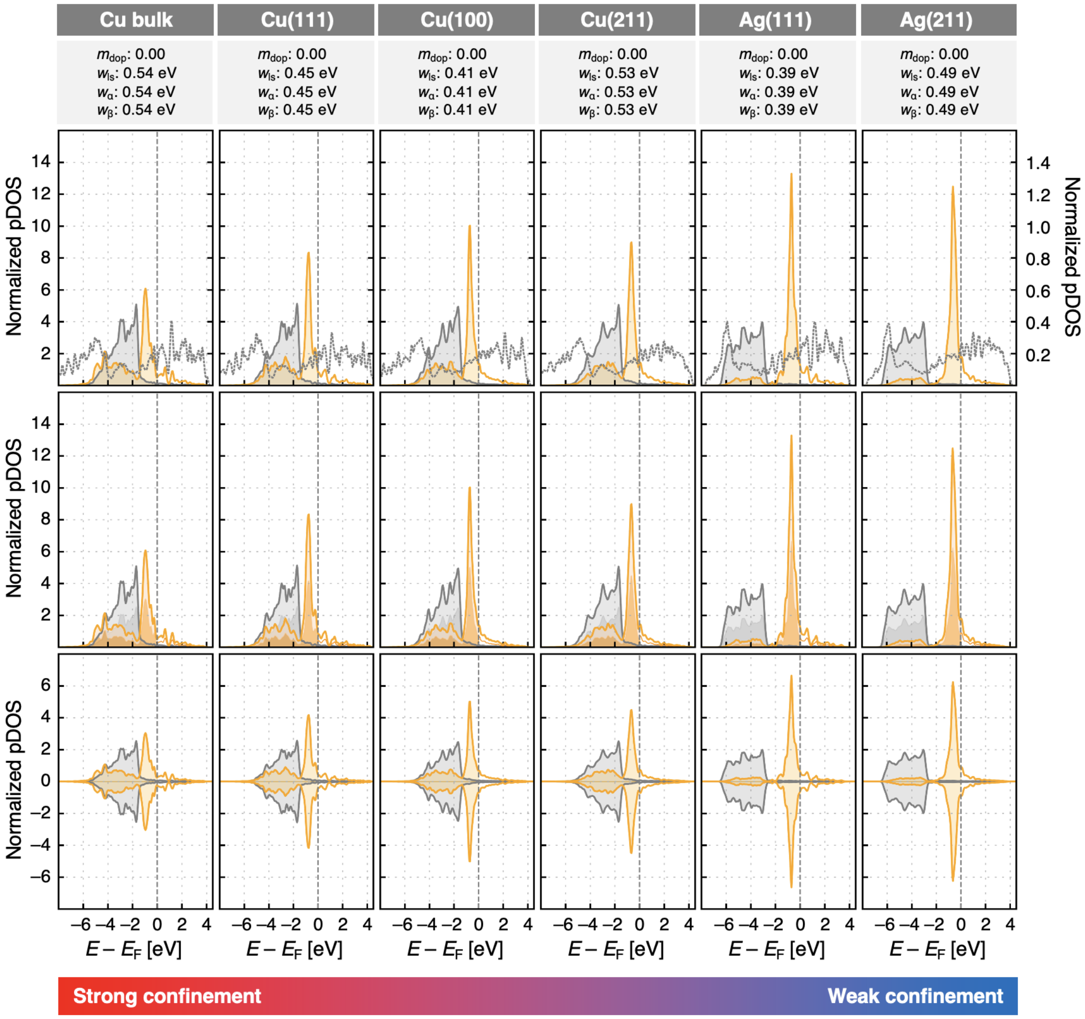


**Figure S7:** Projected densities of states (pDOS) for the most stable spin configuration ($m_{dop}$) of the **dopant Rh** (orange) and the Cu and Ag host surfaces (gray), normalized per atom. The width of the main dopant $d$-band feature, measured as the full width at half maximum in the spin-unpolarized ($w_{\mathrm{ls}}$), alpha ($w_\alpha$), and beta ($w_\beta$) spin channels, tends to decrease with reduced dopant confinement. Shaded areas indicate $d$-band filling up to the Fermi level ($E_{\mathrm{F}}$, gray dashed vertical line). **Top panels**: Solid lines represent the pDOS of dopant and host $d$-states; dotted lines show host $s$-states. The largely unaltered shape of the host $s$-state pDOS indicates minimal mixing with dopant $d$-states. **Mid panels**: Cumulative pDOS of dopant and host $d$-states including both alpha and beta spin channels. Beta contributions (light gray, light orange) are stacked atop alpha contributions (dark gray, dark orange), separated by a line. **Bottom panels**: Spin-resolved pDOS of dopant and host $d$-states, with alpha (positive) and beta (negative) spin contributions shown with opposite signs.

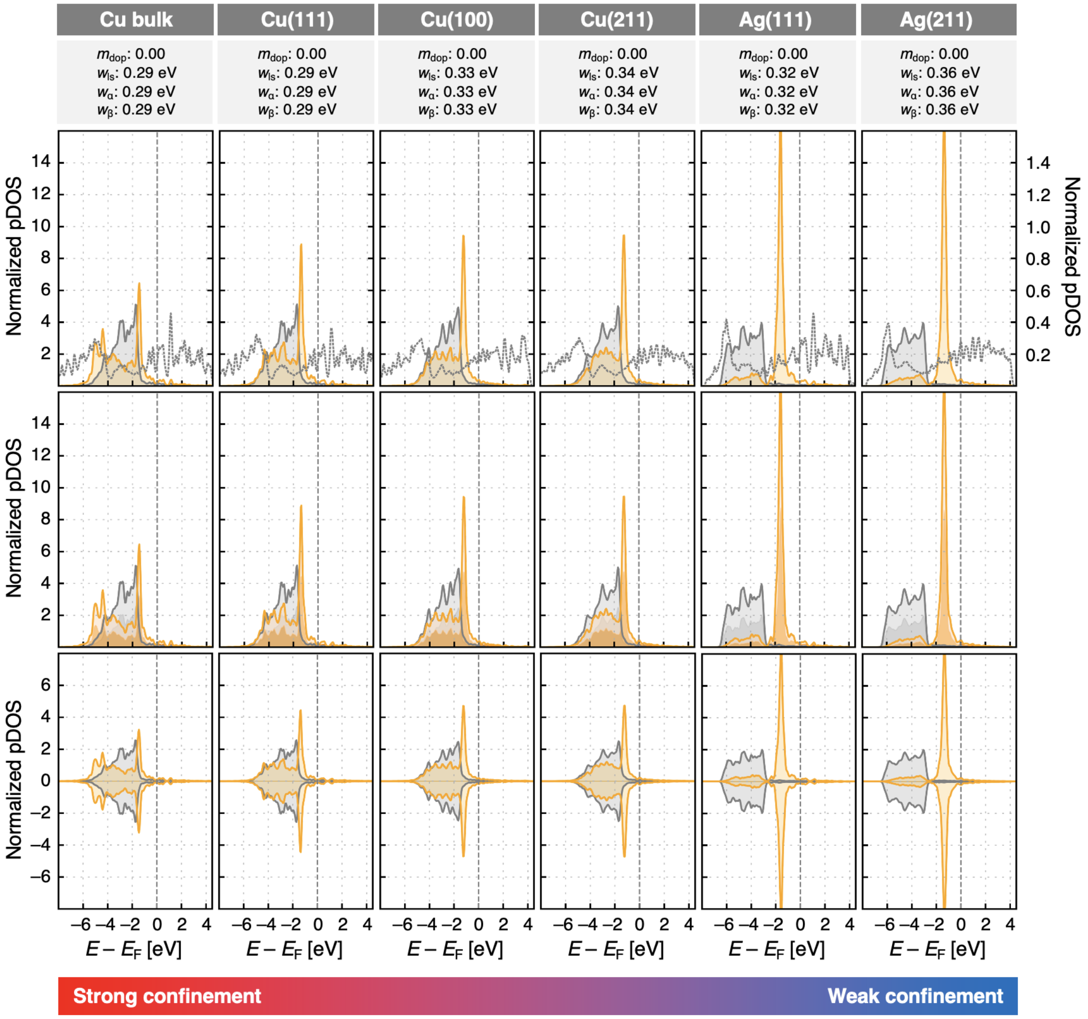


**Figure S8:** Projected densities of states (pDOS) for the most stable spin configuration ($m_{dop}$) of the **dopant Pd** (orange) and the Cu and Ag host surfaces (gray), normalized per atom. The width of the main dopant $d$-band feature, measured as the full width at half maximum in the spin-unpolarized ($w_{\text{ls}}$), alpha ($w_\alpha$), and beta ($w_\beta$) spin channels, tends to decrease with reduced dopant confinement. Shaded areas indicate $d$-band filling up to the Fermi level ($E_{\text{F}}$, gray dashed vertical line). **Top panels**: Solid lines represent the pDOS of dopant and host $d$-states; dotted lines show host $s$-states. The largely unaltered shape of the host $s$-state pDOS indicates minimal mixing with dopant $d$-states. **Mid panels**: Cumulative pDOS of dopant and host $d$-states including both alpha and beta spin channels. Beta contributions (light gray, light orange) are stacked atop alpha contributions (dark gray, dark orange), separated by a line. **Bottom panels**: Spin-resolved pDOS of dopant and host $d$-states, with alpha (positive) and beta (negative) spin contributions shown with opposite signs.

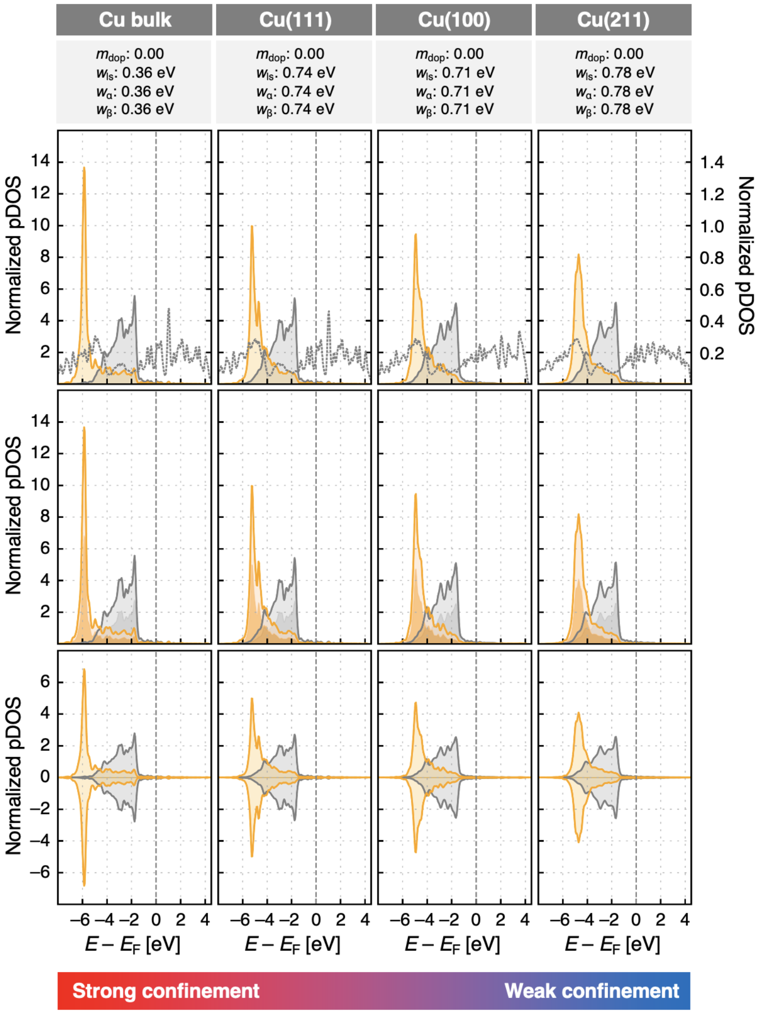


**Figure S9:** Projected densities of states (pDOS) for the most stable spin configuration ($m_{dop}$) of the **dopant Ag** (orange) and the Cu host surfaces (gray), normalized per atom. The width of the main dopant $d$-band feature, measured as the full width at half maximum in the spin-unpolarized ($w_{\mathrm{ls}}$), alpha ($w_{\alpha}$), and beta ($w_{\beta}$) spin channels, tends to decrease with reduced dopant confinement. Shaded areas indicate $d$-band filling up to the Fermi level ($E_{\mathrm{F}}$, gray dashed vertical line). **Top panels**: Solid lines represent the pDOS of dopant and host $d$-states; dotted lines show host $s$-states. The largely unaltered shape of the host $s$-state pDOS indicates minimal mixing with dopant $d$-states. **Mid panels**: Cumulative pDOS of dopant and host $d$-states including both alpha and beta spin channels. Beta contributions (light gray, light orange) are stacked atop alpha contributions (dark gray, dark orange), separated by a line. **Bottom panels**: Spin-resolved pDOS of dopant and host $d$-states, with alpha (positive) and beta (negative) spin contributions shown with opposite signs.

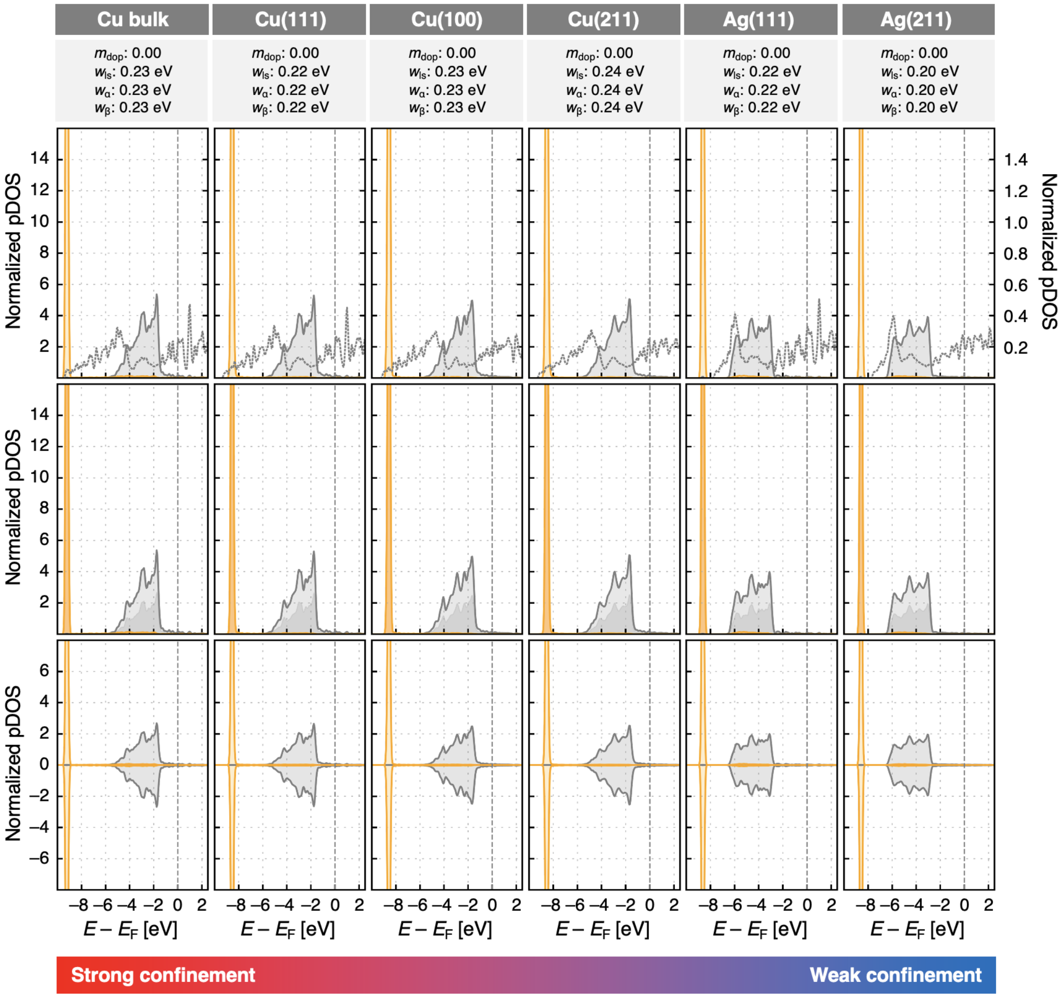


**Figure S10:** Projected densities of states (pDOS) for the most stable spin configuration ($m_{dop}$) of the **dopant Cd** (orange) and the Cu and Ag host surfaces (gray), normalized per atom. The width of the main dopant $d$-band feature, measured as the full width at half maximum in the spin-unpolarized ($w_{\text{ls}}$), alpha ($w_{\alpha}$), and beta ($w_{\beta}$) spin channels, tends to decrease with reduced dopant confinement. Shaded areas indicate $d$-band filling up to the Fermi level ($E_{\text{F}}$, gray dashed vertical line). **Top panels**: Solid lines represent the pDOS of dopant and host $d$-states; dotted lines show host $s$-states. The largely unaltered shape of the host $s$-state pDOS indicates minimal mixing with dopant $d$-states. **Mid panels**: Cumulative pDOS of dopant and host $d$-states including both alpha and beta spin channels. Beta contributions (light gray, light orange) are stacked atop alpha contributions (dark gray, dark orange), separated by a line. **Bottom panels**: Spin-resolved pDOS of dopant and host $d$-states, with alpha (positive) and beta (negative) spin contributions shown with opposite signs.

# S3 Molecular Orbital Perspective on Dopant and Host State Mixing

The limited mixing between dopant and host $d$-states, particularly for late TM dopants in coinage metal hosts, can be understood through a molecular orbital (MO) framework (Figure S11). While energetic alignment and spatial overlap are commonly considered primary factors in hybridization strength,[S10,S11] the occupation of these states plays an equally important role.

When both the dopant and host have filled or nearly filled $d$-bands, hybridization leads to the formation of fully filled bonding orbitals and mostly or fully populated antibonding orbitals. Bonding orbitals offer stabilization, while antibonding orbitals, when populated, introduce an energy penalty. For late TM dopants, which fill both bonding and antibonding states, strong hybridization results in a net energy loss. Consequently, state mixing is energetically unfavorable and avoided. This MO-based rationale explains why late TM dopants retain narrow, free-atom-like $d$-bands, even when their $d$-states align more closely with those of the host. These insights expand the prevailing view of dopant–host mixing by highlighting the dopant's $d$-electron count as a key factor. While energy mismatches and spatial confinement influence hybridization, the number of $d$-electrons in late TM dopants further impedes state mixing.

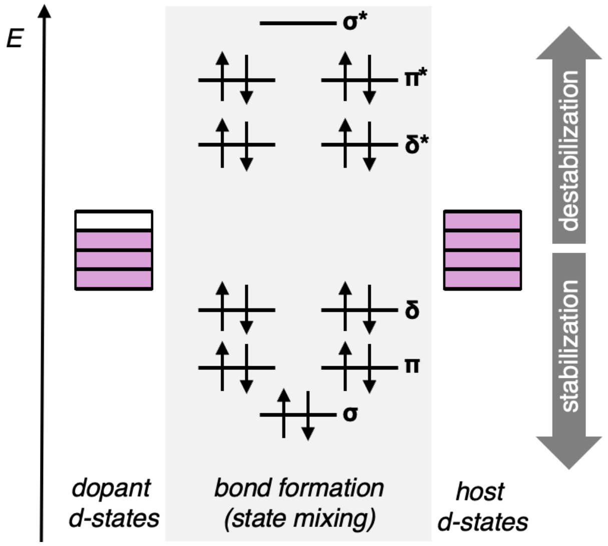


**Figure S11:** Schematic molecular orbital (MO) diagram illustrating the energetics of dopant–host $d$-state hybridization. When late TM dopants with filled $d$-orbitals interact with coinage metal hosts that also have filled $d$-bands, the formation of occupied antibonding states leads to a net energy penalty. Consequently, hybridization is energetically disfavored, and dopant $d$-states remain electronically decoupled and free-atom-like.

## S4 Periodic Trends in Dopant Protrusion

To investigate the influence of dopant–host interactions on the surface morphology of SAAs, we analyze the vertical displacement (protrusion) of $4d$ TM dopants embedded in Cu(111) and Ag(111) surfaces. This protrusion, defined as the height difference between the dopant atom and the host surface plane, arises from two main factors: the strength of dopant–host bonding and the steric strain introduced by size mismatch between the dopant and the host lattice.

Figure S12 illustrates the periodic trend in dopant protrusion across the $4d$ series, with values reported in Table S7. In both Cu(111) and Ag(111), early and late TM dopants tend to protrude more strongly, while central elements often lie nearly level with the surface. For example, Y and Cd protrude by 92 and 72pm, respectively, in Cu(111), while Ru is slightly recessed by –4 pm. In Ag(111), where the larger host lattice generally reduces the size mismatch, protrusion magnitudes are smaller overall, but the trend remains consistent.

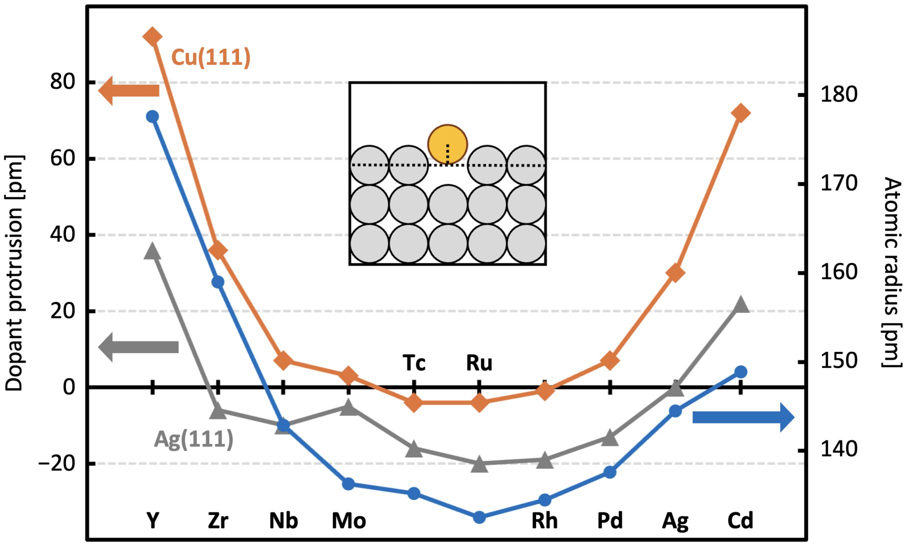


**Figure S12:** Periodic trends in the vertical displacement (protrusion) of $4d$ TM dopants relative to the surface level in Cu(111) (orange diamonds) and Ag(111) (gray triangles), reported in pm. Atomic radii (blue circles), defined as half the interatomic distance in the elemental bulk, are shown for comparison. Protrusion generally correlates with atomic size: early and late TMs exhibit strong protrusion, while central TMs are often recessed. Protrusion is more pronounced in the Cu host due to greater size mismatch. At a given atomic radius, late TMs protrude more than early TMs, suggesting a secondary effect beyond size alone.

**Table S7:** Vertical displacement (protrusion; $p$) values for $4d$ transition metal dopants embedded in Cu(111) and Ag(111), alongside the atomic radii ($r_{\mathrm{atom}}$). All values are reported in pm. Atomic radii are defined as half the interatomic distance in the elemental bulk.

| TM | $p_{\text{dop-Cu(111)}}$ | $p_{\text{dop-Ag(111)}}$ | $r_{\text{atom}}$ |
|---|---|---|---|
| Y | 92 | 36 | 178 |
| Zr | 36 | – | 159 |
| Nb | 7 | −10 | 143 |
| Mo | 3 | −5 | 136 |
| Tc | −4 | −16 | 135 |
| Ru | −4 | −20 | 133 |
| Rh | −1 | −19 | 135 |
| Pd | 7 | −13 | 138 |
| Ag | 30 | 0 | 145 |
| Cd | 72 | 22 | 149 |

The dominant factor governing protrusion is atomic size, as shown by the correlation between dopant atomic radii and their vertical displacement. Larger dopants generally protrude more. However, deviations from this size-based trend indicate a secondary effect: specifically, late TMs protrude more than early TMs of similar atomic size. For instance, Ag (atomic radius 145 pm) embedded in Cu(111) protrudes by 30 pm, while Nb (143 pm) protrudes by only 7 pm. This suggests that size alone does not fully capture the observed morphological trends. The disproportionate protrusion of late TMs becomes particularly evident when comparing the earliest and latest elements in the series. Y (178 pm) and Cd (149 pm) differ in atomic radius by 29 pm, while Cd is only 16 pm larger than Ru (133pm). That is, 36% of the Y–Ru size difference ($16/45 = 0.36$). Yet, Cd protrudes by 72 pm, which is 79% of the full protrusion range from Ru (–4 pm) to Y (92 pm): $(72+4)/(92+4) = 0.79$. This overproportional response suggests that late TM dopants exhibit an enhanced tendency to relax out of the surface compared to what would be expected from size alone.

We propose that this asymmetry arises from differences in hybridization. As explained

in Section S3 of the Supporting Information, late transition metals are expected to avoid hybridization with the host due to their filled or nearly filled $d$-states. This reduces dopant–host bonding and lowers the energetic penalty for vertical displacement from the surface plane, when such displacement alleviates steric strain caused by size mismatch. Together, these findings indicate that both structural (size mismatch) and electronic (hybridization) factors govern dopant positioning in SAAs, with atomic size serving as the primary driver of protrusion. Secondary deviations from this size-based trend arise from differences in dopant–host bonding strength, which can facilitate greater protrusion when needed to relieve steric strain.

## S5 Quantification of Dopant Confinement

To quantify the degree of confinement experienced by dopant atoms in different host environments, we introduce two complementary metrics: (1) a volume-normalized discrete coordination number, $CN^*$, and (2) a continuous coordination number, $CN^\dagger$.

**1. Volume-Normalized Coordination Number ($CN^*$)**

The discrete coordination number ($CN$) counts the number of host atoms directly bonded to a dopant in the environment $i$, as shown in Table S8. To enable comparison across hosts with different lattice parameters, we normalize $CN$ by the volume of a cube defined by the host's bulk interatomic distance, $d_{\text{host}}$:

$$CN^*_{i,\text{host}} = \frac{CN_i}{d^3_{\text{host}}} \tag{S1}$$

The values of $d_{\text{host}}$ are obtained via equation-of-state calculations using the optB86b-vdW functional: $d_{\text{Cu}} = 2.55$ Å, $d_{\text{Ag}} = 2.89$ Å, and $d_{\text{Au}} = 2.91$ Å.

**2. Continuous Coordination Number ($CN^\dagger$)**

We also define a continuous coordination number, $CN^\dagger$, inspired by expressions more commonly used in molecular dynamics simulations. This metric accounts for both the number and proximity of neighboring atoms:

$$CN^\dagger_{i,\text{host}} = \sum^{CN_i} \frac{1}{1 + \left(\frac{d_{\text{host}}}{r_0}\right)^6} \tag{S2}$$

Here, $r_0$ is a reference distance, set to the average of the bulk interatomic distances of Cu and Ag ($r_0 = 2.72$ Å), and the exponent is set to 6. This functional form ensures a smooth decay in contribution from atoms further from the dopant.

As shown in Table S8 and Figure S13, both metrics yield the same relative ordering of dopant environments and show nearly identical trends. This agreement demonstrates the robustness of our confinement descriptors and supports the ordering of environments

discussed in the main text.

**Table S8:** Confinement metrics for dopants in various host environments. Reported values include the bulk interatomic distance of the host metal ($d_{\text{host}}$, in Å), the discrete coordination number ($CN$), the volume-normalized coordination number ($CN^*$), and the continuous coordination number ($CN^\dagger$). These metrics demonstrate a consistent decrease in dopant confinement from Cu bulk to Ag(211).

| | Cu bulk | Cu(111) | Cu(100) | Cu(211) | Ag(111) | Ag(100) | Ag(211) | Au(100) |
|---|---|---|---|---|---|---|---|---|
| $d_{\text{host}}$ | 2.55 | 2.55 | 2.55 | 2.55 | 2.89 | 2.89 | 2.89 | 2.91 |
| $CN$ | 12 | 9 | 8 | 7 | 9 | 8 | 7 | 8 |
| $CN^*$ | 0.72 | 0.54 | 0.48 | 0.42 | 0.37 | 0.33 | 0.29 | 0.32 |
| $CN^\dagger$ | 7.15 | 5.36 | 4.76 | 4.17 | 3.69 | 3.28 | 2.87 | 3.20 |

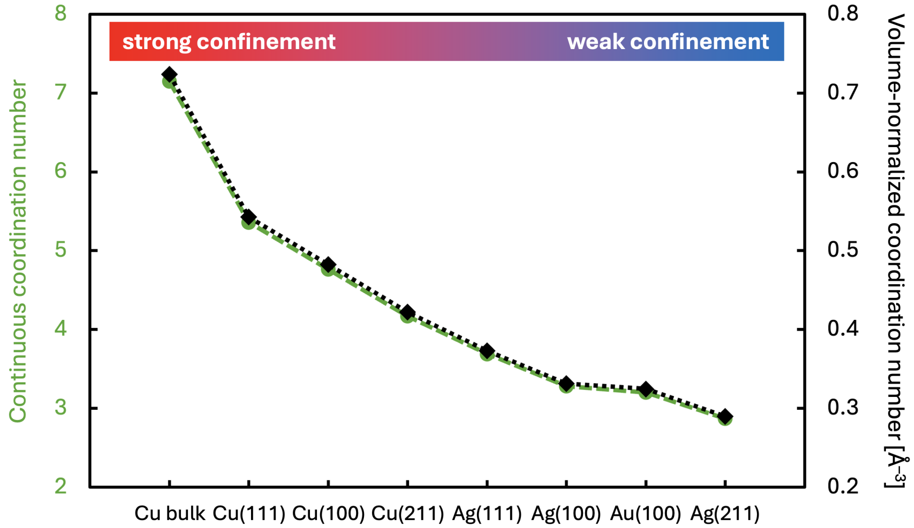


**Figure S13:** Comparison of dopant confinement metrics for all investigated environments. Both the volume-normalized coordination number ($CN^*$) and the continuous coordination number ($CN^\dagger$) yield the same qualitative ordering of confinement across host surfaces. The nearly identical trends support the robustness of these descriptors.

# S6 Separating Coordination, Spacing, and Finite-Size Effects

To decouple the effects of local coordination symmetry and dopant–host spacing on the electronic structures of embedded transition metals in SAAs, we calculate the electronic structures of Zr and Mo embedded in different Cu surface facets at increasing lattice constants and compare them to the corresponding Ag surfaces. These calculations are carried out on the (111), (100), and (211) surface facets, which provide different local coordination environments. To independently modulate dopant–host spacing while maintaining symmetry, we incrementally expanded the Cu lattice by 3%, 6.6%, and 13.3%, the last of which corresponds to the lattice constant of Ag (4.08 Å). This approach allows us to distinguish the influence of dopant–host spacing from that of coordination symmetry.

Figure S14 (left panel) and Table S9 show that increasing the lattice constant consistently reduces the $d$-band width of Zr dopants across all Cu surface facets. This trend reflects a decrease in $d$-state overlap between the dopant and its surrounding host atoms as the dopant–host distance increases. Notably, when Cu surfaces are expanded to the Ag lattice constant, the Zr $d$-band widths in Cu become narrower than those in Ag. This occurs because Cu atoms are smaller than Ag atoms. At identical lattice constants, the smaller Cu atoms provide more space around the dopant than the larger Ag atoms, resulting in reduced dopant confinement in the Cu host. Consequently, the $d$-band widths in Cu not only approach but even surpass those observed in corresponding Ag surfaces. The facet-specific ordering of $d$-band widths (Cu(111)   Cu(100)   Cu(211)) remains intact, underscoring the influence of coordination symmetry, although its impact diminishes as dopant–host spacing increases.

To probe the influence of dopant–host distance and local coordination on magnetic properties, specifically magnetization and the relative energy difference between high-spin and low-spin states, we calculate the spin polarization of Mo dopants in the same set of surfaces. As shown in Figure S14 (right panel), Table S10, and Table S11, both the dopant mag-

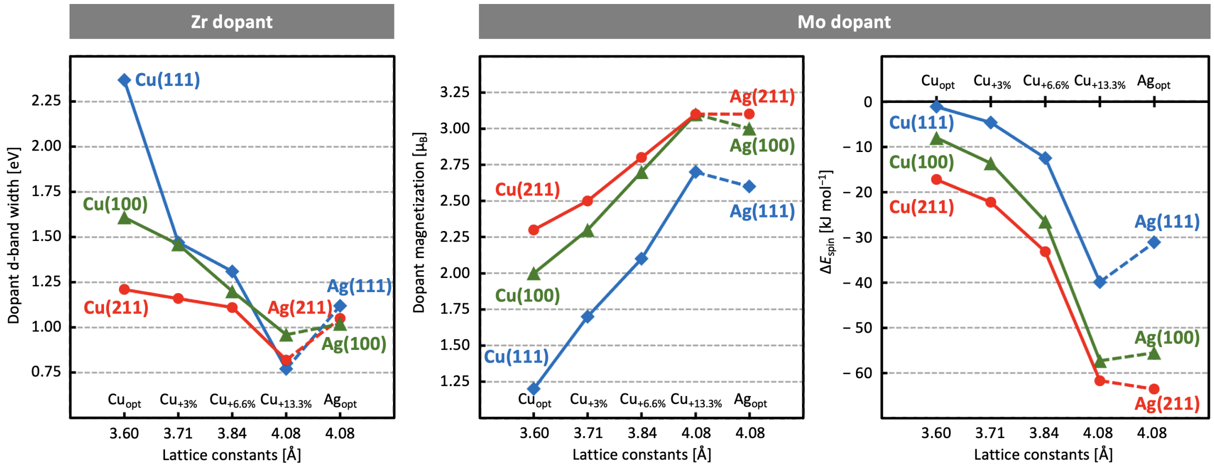


**Figure S14:** Dependence of dopant electronic structure on coordination symmetry and dopant–host distance, analyzed using the (111) (blue diamonds), (100) (green triangles), and (211) (red circles) surface facets. Dopant–host distance is varied by expanding the Cu lattice constant from its equilibrium value (3.60 Å) to 3.71 Å (+3%), 3.84 Å (+6.6%), and 4.08 Å (+13.3%), the latter matching the equilibrium lattice constant of Ag. Left: For Zr in Cu, the *d*-band width decreases with increasing lattice constant, approaching or even dropping below the values observed in the corresponding Ag surfaces, consistent with reduced confinement. While facet-dependent symmetry effects also influence the *d*-band width, their impact diminishes at larger dopant–host distances. Right: Similarly, both the dopant magnetization and the energetic stabilization of the high-spin state for Mo, $\Delta E_{\text{spin}}$, increase with reduced confinement.

netization and the energetic stabilization of the high-spin state increase with dopant–host distance. For example, on the Cu(100) surface, the magnetization rises from 2.0 $\mu_\text{B}$ at the equilibrium lattice constant (3.60 Å) to 3.1 $\mu_\text{B}$ at the Ag lattice constant (4.08 Å), accompanied by a substantial increase in high-spin stabilization from –8 to –57 kJ mol$^{-1}$. At the Ag lattice constant, the magnetization and spin-state stabilization in Cu exceed those observed on the corresponding Ag surfaces, reflecting the reduced confinement of the dopant due to the smaller Cu atomic size.

While all surface facets follow this general trend, the absolute values remain facet dependent, with lower-coordination environments promoting stronger magnetization and greater stabilization of the high-spin state. However, the impact of the facet weakens as dopant–host spacing increases and the effect of direct coordination diminishes.

Together, these results demonstrate that dopant–host distance and local coordination symmetry exert partially independent effects on the electronic and magnetic properties of

**Table S9:** Dopant $d$-band widths (in eV) for Zr embedded in Cu surfaces with (111), (100), and (211) facets at different lattice constants. Lattice constants are set to 3.60 Å (0%), 3.71 Å (+3%), 3.84 Å (+6.6%), and 4.08 Å (+13.3%) to reflect variations in coordination and dopant–host distance and their effects on the dopant's electronic structure.

| | $Cu_{opt}$ | $Cu_{+3\%}$ | $Cu_{+6.6\%}$ | $Cu_{+13.3\%}$ | $Ag_{opt}$ |
|---|---|---|---|---|---|
| Cu(111) | 2.37 | 1.47 | 1.31 | 0.77 | 1.12 |
| Cu(100) | 1.21 | 1.16 | 1.11 | 0.82 | 1.05 |
| Cu(211) | 1.61 | 1.46 | 1.20 | 0.96 | 1.02 |

SAAs. Coordination symmetry, defined by the surface facet, strongly influences the electronic structure of dopants, but its impact gradually diminishes as the dopant–host distance increases. The effects of dopant–host distance and coordination environment are of comparable magnitude, indicating that both the surface facet and host metal identity serve as effective levers to tune the electronic structure of active dopant sites.

**Table S10:** Dopant magnetization (in $\mu_B$) for Mo embedded in Cu surfaces with (111), (100), and (211) facets at different lattice constants. Lattice constants are set to 3.60 Å (0%), 3.71 Å (+3%), 3.84 Å (+6.6%), and 4.08 Å (+13.3%) to reflect variations in coordination and dopant–host distance and their effects on the dopant's spin state.

| | $Cu_{opt}$ | $Cu_{+3\%}$ | $Cu_{+6.6\%}$ | $Cu_{+13.3\%}$ | $Ag_{opt}$ |
|---|---|---|---|---|---|
| Cu(111) | 1.2 | 1.7 | 2.1 | 2.7 | 2.6 |
| Cu(100) | 2.0 | 2.3 | 2.7 | 3.1 | 3.0 |
| Cu(211) | 2.3 | 2.5 | 2.8 | 3.1 | 3.1 |

**Table S11:** Relative energies (in kJ $mol^{-1}$) of the high-spin state compared to the low-spin state for Mo embedded in Cu surfaces with (111), (100), and (211) facets at different lattice constants. Lattice constants are set to 3.60 Å (0%), 3.71 Å (+3%), 3.84 Å (+6.6%), and 4.08 Å (+13.3%) to reflect variations in coordination and dopant–host distance and their effects on spin-state stability.

| | $Cu_{opt}$ | $Cu_{+3\%}$ | $Cu_{+6.6\%}$ | $Cu_{+13.3\%}$ | $Ag_{opt}$ |
|---|---|---|---|---|---|
| Cu(111) | −1 | −5 | −12 | −40 | −31 |
| Cu(100) | −8 | −14 | −27 | −57 | −55 |
| Cu(211) | −17 | −22 | −33 | −62 | −64 |

To assess finite-size effects, we calculated the electronic structures of Zr, Mo, and Rh dopants in Cu(111) and Ag(111) using larger 5×5×6 surface slabs. Table S12 shows that the dopant $d$-band centers, occupations, and dopant–host distances remain similar to the 3×3×6 slabs used throughout this work. Variations in $d$-band width are small relative to the periodic trends discussed in the main text. For example, for the crucial case of Zr (a large dopant) in Cu(111) (a dense surface with short interatomic distances), the $d$-band width decreases only slightly from 2.37 eV in the 3×3×6 slab to 2.24 eV in the 5×5×6 slab. This is a small change compared to the broadening observed across the dopant series. We therefore conclude that the periodic trends derived from the 3×3×6 models are robust.

**Table S12:** Electronic structure descriptors for the dopants Zr, Mo, and Rh in Cu(111) and Ag(111) obtained with the standard slab size used in this work (3×3×6) and a larger simulation cell (5×5×6) show no significant finite-size effects. Reported quantities are the center of the main dopant $d$-band feature ($c_{\text{dop}}$), the dopant $d$-band filling up to the Fermi level ($f_{\text{dop}}$), the width of the main dopant $d$-band feature measured as the full width at half maximum (FWHM, $w_{\text{dop}}$), and the average dopant–host distance ($d_{\text{dop-host}}$). Energies are reported in eV, occupations in electrons, and distances in pm. Variations with slab size are small compared to the systematic trends discussed in the main text, confirming that conclusions derived from the 3×3×6 slab models are robust.

| SAA | Slab size | $c_{\text{dop}}$ | $f_{\text{dop}}$ | $w_{\text{dop}}$ | $d_{\text{dop-host}}$ |
|---|---|---|---|---|---|
| ZrCu(111) | 3×3×6 | 1.07 | 3.01 | 2.37 | 262.3 |
| | 5×5×6 | 1.08 | 2.96 | 2.24 | 265.5 |
| ZrAg(111) | 3×3×6 | 0.87 | 2.76 | 1.12 | 289.4 |
| | 5×5×6 | 0.82 | 2.75 | 1.03 | 290.1 |
| MoCu(111) | 3×3×6 | 0.47 | 5.47 | 1.24 | 256.3 |
| | 5×5×6 | 0.46 | 5.45 | 1.53 | 258.1 |
| MoAg(111) | 3×3×6 | 0.23 | 5.24 | 0.85 | 285.9 |
| | 5×5×6 | 0.25 | 5.23 | 0.96 | 285.2 |
| RhCu(111) | 3×3×6 | -0.33 | 8.69 | 0.45 | 256.4 |
| | 5×5×6 | -0.34 | 8.67 | 0.47 | 257.1 |
| RhAg(111) | 3×3×6 | –0.48 | 8.65 | 0.39 | 285.9 |
| | 5×5×6 | –0.51 | 8.65 | 0.43 | 284.8 |

# S7 Machine Learning Model for Predicting Dopant *d*-Band Widths

A machine learning (ML) workflow is developed to construct sparse, interpretable models for predicting the $d$-band width of the dopant atom ($w_{\mathrm{dopant}}$) using four physically meaningful input features.

- Dopant atomic radius, $r_{\mathrm{d}}$
- Host atomic radius, $r_{\mathrm{h}}$
- Formal number of dopant $d$-electrons, $n_{\mathrm{d}}$
- Dopant coordination number, $CN_{\mathrm{d}}$

**Data Preprocessing**

Raw input features are first centered and normalized by subtracting the mean and dividing by the standard deviation.

$$r_{\mathrm{d}}^{*} = \frac{r_{\mathrm{d}} - 145.75}{15.29} \tag{S3}$$

$$r_{\mathrm{h}}^{*} = \frac{r_{\mathrm{h}} - 133.37}{7.87} \tag{S4}$$

$$n_{\mathrm{d}}^{*} = \frac{n_{\mathrm{d}} - 5.50}{2.29} \tag{S5}$$

$$CN_{\mathrm{d}}^{*} = \frac{CN_{\mathrm{d}} - 8.67}{1.70} \tag{S6}$$

Here, an asterisk denotes a normalized feature. A library of compound features is constructed from these standardized inputs, including:

- Squared and inverse terms (e.g., $(r^*_{\mathrm{d}})^2$, $1/n^*_{\mathrm{d}}$)
- Cross-terms (e.g., $r^*_{\mathrm{d}} \cdot CN^*_{\mathrm{d}}$)
- Linear combinations (e.g., $r^*_{\mathrm{d}} - n^*_{\mathrm{d}}$)

**Model Training and Feature Selection**

Lasso regression (least absolute shrinkage and selection operator), a linear model with L1 regularization, is employed with 10-fold cross-validation to train a model on the full set of compound features. Features with nonzero coefficients are identified as the most relevant descriptors selected by the model. A second, reduced model is then trained using only the four compound features with the largest absolute Lasso coefficients. The compound feature selection procedure is further constrained to include all raw input features.

The resulting top-4 model model is:

$$\begin{aligned} w^{\text{top-4}}_{\text{dopant}} = \quad & 0.1395 \cdot (r^*_{\mathrm{d}} \cdot CN^*_{\mathrm{d}}) + 0.18982 \cdot (r^*_{\mathrm{d}} - n^*_{\mathrm{d}}) \\ & - 0.1454 \cdot (n^*_{\mathrm{d}} - CN^*_{\mathrm{d}}) - 0.0585 \cdot (r^*_{\mathrm{h}} \cdot r^*_{\mathrm{d}}) \end{aligned} \tag{S7}$$

The resulting all-feature model model is:

$$\begin{aligned} w^{\text{all-features}}_{\text{dopant}} = & -0.0035 \cdot r^*_{\mathrm{d}} \cdot r^*_{\mathrm{d}} + 0.0364 \cdot CN^*_{\mathrm{d}} \cdot CN^*_{\mathrm{d}} + 0.0027 \cdot 1/r^*_{\mathrm{d}} \\ & + 0.0252 \cdot 1/CN^*_{\mathrm{d}} - 0.0502 \cdot r^*_{\mathrm{h}} \cdot r^*_{\mathrm{d}} + 0.0260 \cdot r^*_{\mathrm{h}} \cdot n^*_{\mathrm{d}} \\ & + 0.0661 \cdot r^*_{\mathrm{d}} \cdot CN^*_{\mathrm{d}} - 0.0400 \cdot n^*_{\mathrm{d}} \cdot CN^*_{\mathrm{d}} - 0.0008 \cdot r^*_{\mathrm{h}}/r^*_{\mathrm{d}} \\ & - 0.0164 \cdot r^*_{\mathrm{h}}/CN^*_{\mathrm{d}} + 0.0138 \cdot r^*_{\mathrm{d}}/CN^*_{\mathrm{d}} - 0.0107 \cdot n^*_{\mathrm{d}}/CN^*_{\mathrm{d}} \\ & - 0.0391 \cdot (r^*_{\mathrm{h}} + n^*_{\mathrm{d}}) + 0.1921 \cdot (r^*_{\mathrm{d}} - n^*_{\mathrm{d}}) - 0.0637 \cdot (n^*_{\mathrm{d}} - CN^*_{\mathrm{d}}) \end{aligned} \tag{S8}$$

## Model Performance and Transferability

Figure S15 presents parity plots comparing predicted versus DFT-calculated $d$-band widths for low-spin configurations. The left panel shows the model trained using all 15 non-zero compound features, and the central panel shows the model using the top four compound features. The full model achieves a root-mean-square deviation (RMSD) of 0.17 eV and explains 92% of the variance, while the reduced four-feature model attains an RMSD of 0.22 eV and explains 86% of the variance in dopant $d$-band widths. While the reduced model captures the correct trends and provides semi-quantitative agreement with DFT, and the full model achieves nearly quantitative accuracy, the resulting equations do not yield easily interpretable structure–property relationships. However, the performance of both models confirms that the four raw inputs encode most of the variation in $d$-band widths, supporting the physical descriptors and guiding principles discussed in the main text.

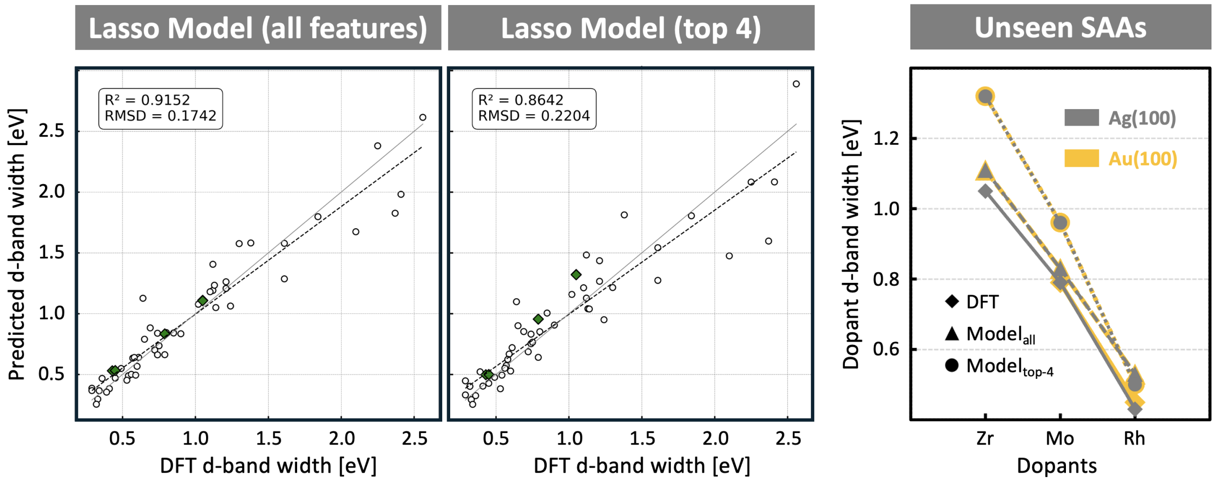


**Figure S15:** DFT-calculated dopant $d$-band widths (diamonds) for Zr, Mo, and Rh in Ag(100) (gray) and Au(100) (gold) surfaces, compared with predictions from a Lasso-based ML model using either all features (triangles) or the top four compound descriptors (circles). Left: Parity plot showing model performance across the training set (excluding Ag(100) and Au(100)). The full-feature model captures 92% of the variance with an RMSD of 0.17 eV, while the top-4 model explains 86% with an RMSD of 0.22 eV. Diamonds represent test data (Ag(100) and Au(100)); circles denote training data. The solid gray lines indicate perfect correlation (predicted $d$-band width = DFT $d$-band width), and the dashed black lines show the linear fits to the model predictions. Right: $d$-band widths decrease from early to late TMs, with nearly identical DFT values for Ag(100) and Au(100), consistent with their similar lattice constants. The full-feature model achieves an average deviation of 0.06 eV, while the top-4 model captures the trend semi-quantitatively.

To assess the robustness of the ML models, we performed leave-p-out validation tests by training on subsets of the dopants and predicting the left-out data. For the full model, the RMSE increases gradually from 0.17 eV for the full training set to 0.19, 0.23, 0.27, 0.26, 0.32, and 0.30 eV when leaving out one to six dopants, respectively. Interestingly, the reduced top-4 model shows even greater stability: although its training error is somewhat larger (0.22 eV), the RMSE decreases to 0.17 eV for leave-one-out and then remains nearly constant as more data are removed (0.21, 0.22, 0.23, 0.24, 0.24, and 0.24 eV). These results demonstrate that both models retain predictive accuracy even when a sizable fraction of the dataset is left out. This further underscores the robustness and transferability of these models, and in particular of the compact top-4 model.

To further evaluate the generality and predictive power of the models, we test them on two new sets of SAA systems not included during model development: Zr, Mo, and Rh dopants in Ag(100), a surface facet absent from the training set, and the same dopants in Au(100), representing a completely new host metal. These systems are selected to investigate the transferability of the models. The full-feature model predicts the dopant $d$-band widths for these systems with an average deviation of only 0.06 eV from the DFT reference values, demonstrating quantitative agreement, as shown in the right panel of Figure S15. In addition, the DFT-based and predicted $d$-band widths for a given dopant in Ag(100) and Au(100) are nearly identical, differing by no more than 0.02 eV. Among the primary factors identified by our design guidelines, the only one that changes between Ag(100) and Au(100) is the interatomic distance between the host atoms, and this change is minimal, resulting in a correspondingly minute variation in dopant $d$-band width. These results confirm the transferability of the models and their broader applicability to other dilute alloy systems. They also underscore that the selected descriptors, derived from our design principles, encode the key physics governing dopant $d$-band widths, even for systems beyond the original training domain.

**Tabulated Results**

Tables S13 to S19 list the input parameters and corresponding DFT and predicted dopant $d$-band widths. The model is also provided as part of the Supporting Information.

**Table S13:** Input features and predicted dopant $d$-band widths for 4$d$ transition metal dopants in bulk Cu. Shown are the raw input parameters: dopant atomic radius ($r_{\mathrm{d}}$) and host atomic radius ($r_{\mathrm{h}}$) in pm, the formal number of dopant $d$-electrons ($n_{\mathrm{d}}$), and the dopant coordination number ($CN_{\mathrm{d}}$). Also listed are the density functional theory-based reference dopant $d$-band width ($w_{\mathrm{dopant}}$), and the $d$-band widths predicted by the full-feature model ($w_{\text{all-features}}$) and the four-feature model ($w_{\text{4-features}}$), all in eV.

| TM | $r_{\mathrm{d}}$ | $r_{\mathrm{h}}$ | $n_{\mathrm{d}}$ | $CN_{\mathrm{d}}$ | $w_{\mathrm{dopant}}$ | $w_{\text{all-features}}$ | $w_{\text{4-features}}$ |
|---|---|---|---|---|---|---|---|
| Y | 180 | 127.8 | 2 | 12 | 2.56 | 2.62 | 2.89 |
| Zr | 160 | 127.8 | 3 | 12 | 2.41 | 1.98 | 2.08 |
| Nb | 146 | 127.8 | 4 | 12 | 2.10 | 1.67 | 1.48 |
| Mo | 139 | 127.8 | 5 | 12 | 0.64 | 1.13 | 1.10 |
| Tc | 136 | 127.8 | 6 | 12 | 0.69 | 0.88 | 0.85 |
| Ru | 134 | 127.8 | 7 | 12 | 0.79 | 0.66 | 0.64 |
| Rh | 134 | 127.8 | 8 | 12 | 0.54 | 0.49 | 0.49 |
| Pd | 137 | 127.8 | 9 | 12 | 0.29 | 0.39 | 0.45 |

**Table S14:** Input features and predicted dopant $d$-band widths for $4d$ transition metal dopants located in the Cu(111) surface. Shown are the raw input parameters: dopant atomic radius ($r_{\mathrm{d}}$) and host atomic radius ($r_{\mathrm{h}}$) in pm, the formal number of dopant $d$-electrons ($n_{\mathrm{d}}$), and the dopant coordination number ($CN_{\mathrm{d}}$). Also listed are the density functional theory-based reference dopant $d$-band width ($w_{\mathrm{dopant}}$), and the $d$-band widths predicted by the full-feature model ($w_{\text{all-features}}$) and the four-feature model ($w_{\text{4-features}}$), all in eV.

| TM | $r_{\mathrm{d}}$ | $r_{\mathrm{h}}$ | $n_{\mathrm{d}}$ | $CN_{\mathrm{d}}$ | $w_{\mathrm{dopant}}$ | $w_{\text{all-features}}$ | $w_{\text{4-features}}$ |
|---|---|---|---|---|---|---|---|
| Y | 180 | 127.8 | 2 | 9 | 2.25 | 2.38 | 2.08 |
| Zr | 160 | 127.8 | 3 | 9 | 2.37 | 1.83 | 1.60 |
| Nb | 146 | 127.8 | 4 | 9 | 1.30 | 1.58 | 1.22 |
| Mo | 139 | 127.8 | 5 | 9 | 1.24 | 1.06 | 0.95 |
| Tc | 136 | 127.8 | 6 | 9 | 0.74 | 0.84 | 0.75 |
| Ru | 134 | 127.8 | 7 | 9 | 0.57 | 0.64 | 0.57 |
| Rh | 134 | 127.8 | 8 | 9 | 0.45 | 0.47 | 0.43 |
| Pd | 137 | 127.8 | 9 | 9 | 0.29 | 0.37 | 0.33 |

**Table S15:** Input features and predicted dopant $d$-band widths for $4d$ transition metal dopants located in the Cu(100) surface. Shown are the raw input parameters: dopant atomic radius ($r_{\mathrm{d}}$) and host atomic radius ($r_{\mathrm{h}}$) in pm, the formal number of dopant $d$-electrons ($n_{\mathrm{d}}$), and the dopant coordination number ($CN_{\mathrm{d}}$). Also listed are the density functional theory-based reference dopant $d$-band width ($w_{\mathrm{dopant}}$), and the $d$-band widths predicted by the full-feature model ($w_{\text{all-features}}$) and the four-feature model ($w_{\text{4-features}}$), all in eV.

| TM | $r_{\mathrm{d}}$ | $r_{\mathrm{h}}$ | $n_{\mathrm{d}}$ | $CN_{\mathrm{d}}$ | $w_{\mathrm{dopant}}$ | $w_{\text{all-features}}$ | $w_{\text{4-features}}$ |
|---|---|---|---|---|---|---|---|
| Y | 180 | 127.8 | 2 | 8 | 1.38 | 1.58 | 1.81 |
| Zr | 160 | 127.8 | 3 | 8 | 1.21 | 1.26 | 1.44 |
| Nb | 146 | 127.8 | 4 | 8 | 1.12 | 1.19 | 1.13 |
| Mo | 139 | 127.8 | 5 | 8 | 0.65 | 0.79 | 0.90 |
| Tc | 136 | 127.8 | 6 | 8 | 0.61 | 0.64 | 0.72 |
| Ru | 134 | 127.8 | 7 | 8 | 0.56 | 0.50 | 0.55 |
| Rh | 134 | 127.8 | 8 | 8 | 0.41 | 0.38 | 0.41 |
| Pd | 137 | 127.8 | 9 | 8 | 0.33 | 0.30 | 0.29 |

**Table S16:** Input features and predicted dopant $d$-band widths for $4d$ transition metal dopants located in the Cu(211) surface. Shown are the raw input parameters: dopant atomic radius ($r_\mathrm{d}$) and host atomic radius ($r_\mathrm{h}$) in pm, the formal number of dopant $d$-electrons ($n_\mathrm{d}$), and the dopant coordination number ($CN_\mathrm{d}$). Also listed are the density functional theory-based reference dopant $d$-band width ($w_\mathrm{dopant}$), and the $d$-band widths predicted by the full-feature model ($w_\text{all-features}$) and the four-feature model ($w_\text{4-features}$), all in eV.

| TM | $r_\mathrm{d}$ | $r_\mathrm{h}$ | $n_\mathrm{d}$ | $CN_\mathrm{d}$ | $w_\mathrm{dopant}$ | $w_\text{all-features}$ | $w_\text{4-features}$ |
|---|---|---|---|---|---|---|---|
| Y | 180 | 127.8 | 2 | 7 | 1.61 | 1.58 | 1.54 |
| Zr | 160 | 127.8 | 3 | 7 | 1.61 | 1.29 | 1.27 |
| Nb | 146 | 127.8 | 4 | 7 | 1.13 | 1.23 | 1.04 |
| Mo | 139 | 127.8 | 5 | 7 | 0.80 | 0.84 | 0.85 |
| Tc | 136 | 127.8 | 6 | 7 | 0.72 | 0.70 | 0.69 |
| Ru | 134 | 127.8 | 7 | 7 | 0.60 | 0.57 | 0.53 |
| Rh | 134 | 127.8 | 8 | 7 | 0.53 | 0.45 | 0.38 |
| Pd | 137 | 127.8 | 9 | 7 | 0.34 | 0.37 | 0.25 |

**Table S17:** Input features and predicted dopant $d$-band widths for $4d$ transition metal dopants located in the Ag(111) surface. Shown are the raw input parameters: dopant atomic radius ($r_\mathrm{d}$) and host atomic radius ($r_\mathrm{h}$) in pm, the formal number of dopant $d$-electrons ($n_\mathrm{d}$), and the dopant coordination number ($CN_\mathrm{d}$). Also listed are the density functional theory-based reference dopant $d$-band width ($w_\mathrm{dopant}$), and the $d$-band widths predicted by the full-feature model ($w_\text{all-features}$) and the four-feature model ($w_\text{4-features}$), all in eV.

| TM | $r_\mathrm{d}$ | $r_\mathrm{h}$ | $n_\mathrm{d}$ | $CN_\mathrm{d}$ | $w_\mathrm{dopant}$ | $w_\text{all-features}$ | $w_\text{4-features}$ |
|---|---|---|---|---|---|---|---|
| Y | 180 | 144.5 | 2 | 9 | 1.84 | 1.80 | 1.81 |
| Zr | 160 | 144.5 | 3 | 9 | 1.12 | 1.41 | 1.48 |
| Nb | 146 | 144.5 | 4 | 9 | 1.10 | 1.18 | 1.21 |
| Mo | 139 | 144.5 | 5 | 9 | 0.85 | 0.84 | 1.01 |
| Tc | 136 | 144.5 | 6 | 9 | 0.74 | 0.66 | 0.83 |
| Ru | 134 | 144.5 | 7 | 9 | 0.59 | 0.50 | 0.67 |
| Rh | 134 | 144.5 | 8 | 9 | 0.39 | 0.36 | 0.52 |
| Pd | 137 | 144.5 | 9 | 9 | 0.32 | 0.26 | 0.40 |

**Table S18:** Input features and predicted dopant $d$-band widths for 4$d$ transition metal dopants located in the Ag(211) surface. Shown are the raw input parameters: dopant atomic radius ($r_{\mathrm{d}}$) and host atomic radius ($r_{\mathrm{h}}$) in pm, the formal number of dopant $d$-electrons ($n_{\mathrm{d}}$), and the dopant coordination number ($CN_{\mathrm{d}}$). Also listed are the density functional theory-based reference dopant $d$-band width ($w_{\mathrm{dopant}}$), and the $d$-band widths predicted by the full-feature model ($w_{\mathrm{all\text{-}features}}$) and the four-feature model ($w_{\mathrm{4\text{-}features}}$), all in eV.

| TM | $r_{\mathrm{d}}$ | $r_{\mathrm{h}}$ | $n_{\mathrm{d}}$ | $CN_{\mathrm{d}}$ | $w_{\mathrm{dopant}}$ | $w_{\mathrm{all\text{-}features}}$ | $w_{\mathrm{4\text{-}features}}$ |
|---|---|---|---|---|---|---|---|
| Y | 180 | 144.5 | 2 | 7 | 1.21 | 1.21 | 1.27 |
| Zr | 160 | 144.5 | 3 | 7 | 1.02 | 1.08 | 1.16 |
| Nb | 146 | 144.5 | 4 | 7 | 1.14 | 1.05 | 1.04 |
| Mo | 139 | 144.5 | 5 | 7 | 0.90 | 0.84 | 0.91 |
| Tc | 136 | 144.5 | 6 | 7 | 0.75 | 0.74 | 0.77 |
| Ru | 134 | 144.5 | 7 | 7 | 0.58 | 0.64 | 0.62 |
| Rh | 134 | 144.5 | 8 | 7 | 0.49 | 0.55 | 0.48 |
| Pd | 137 | 144.5 | 9 | 7 | 0.36 | 0.47 | 0.33 |

**Table S19:** Input features and predicted dopant $d$-band widths for the 4$d$ transition metal dopants Zr, Mo, and Rh located in the Ag(100) and Au(100) surfaces. Shown are the raw input parameters: dopant atomic radius ($r_{\mathrm{d}}$) and host atomic radius ($r_{\mathrm{h}}$) in pm, the formal number of dopant $d$-electrons ($n_{\mathrm{d}}$), and the dopant coordination number ($CN_{\mathrm{d}}$). Also listed are the density functional theory-based reference dopant $d$-band width ($w_{\mathrm{dopant}}$), and the $d$-band widths predicted by the full-feature model ($w_{\mathrm{all\text{-}features}}$) and the four-feature model ($w_{\mathrm{4\text{-}features}}$), all in eV.

| TM | $r_{\mathrm{d}}$ | $r_{\mathrm{h}}$ | $n_{\mathrm{d}}$ | $CN_{\mathrm{d}}$ | $w_{\mathrm{dopant}}$ | $w_{\mathrm{all\text{-}features}}$ | $w_{\mathrm{4\text{-}features}}$ |
|---|---|---|---|---|---|---|---|
| $\mathrm{Zr}_{\mathrm{Ag(100)}}$ | 160 | 144.5 | 3 | 8 | 1.05 | 1.11 | 1.32 |
| $\mathrm{Mo}_{\mathrm{Ag(100)}}$ | 139 | 144.5 | 5 | 8 | 0.79 | 0.83 | 0.96 |
| $\mathrm{Mo}_{\mathrm{Au(100)}}$ | 139 | 144.2 | 5 | 8 | 0.79 | 0.83 | 0.96 |
| $\mathrm{Rh}_{\mathrm{Ag(100)}}$ | 134 | 144.5 | 8 | 8 | 0.43 | 0.53 | 0.50 |
| $\mathrm{Rh}_{\mathrm{Au(100)}}$ | 134 | 144.2 | 8 | 8 | 0.45 | 0.53 | 0.50 |

# S8 Functional Dependence of $d$-Band Width and Spin Polarization

To assess whether the observed trends in dopant electronic structure are artifacts of the chosen exchange–correlation functional, we perform additional density functional theory (DFT) calculations for the three dopants Zr, Mo, and Rh embedded in Cu(111). In addition to the optB86b-vdW functional employed throughout the main text, we test two alternative generalized gradient approximation (GGA) functionals (PBEsol and RPBE) and the meta-GGA functional r$^2$SCAN. These functionals were selected for their known differences in describing lattice constants, surface energetics, and electronic localization.[S12? ,S13]

Figure S16 summarizes the functional dependence of several key electronic descriptors. For non-magnetic dopants (Zr and Rh), the calculated $d$-band centers and widths remain nearly unchanged across all functionals. Table S20 provides the quantitative data, confirming that the impact of enhanced electron localization, as expected for the meta-GGA r$^2$SCAN functional, is minimal for these non-magnetic dopants.

**Table S20:** $d$-Band centers ($c$) and $d$-band widths ($w$), in eV, for Zr and Rh dopants embedded in Cu(111), calculated using four different DFT functionals: optB86b-vdW, PBEsol, RPBE, and r$^2$SCAN. Both quantities remain nearly invariant across functionals, indicating that improved electron localization has little effect on these non-magnetic dopants.

| | $c_{\text{optB86b-vdW}}$ | $w_{\text{optB86b-vdW}}$ | $c_{\text{PBEsol}}$ | $w_{\text{PBEsol}}$ | $c_{\text{RPBE}}$ | $w_{\text{RPBE}}$ | $c_{\text{r}^2\text{SCAN}}$ | $w_{\text{r}^2\text{SCAN}}$ |
|---|---|---|---|---|---|---|---|---|
| Zr | 1.07 | 2.37 | 1.11 | 2.41 | 1.09 | 2.42 | 1.19 | 2.60 |
| Rh | −0.33 | 0.45 | −0.31 | 0.45 | −0.33 | 0.45 | −0.36 | 0.46 |

In contrast, magnetic properties for Mo, which exhibits a high-spin ground state, show a more pronounced functional dependence. Specifically, both the magnetization ($m_{\text{Mo}}$) and the energetic stabilization of the high-spin state ($\Delta E_{\text{spin}}$) increase with functionals that provide stronger electronic localization, following the order optB86b-vdW < PBEsol < RPBE < r$^2$SCAN. This trend aligns with established differences among these functionals. This is most

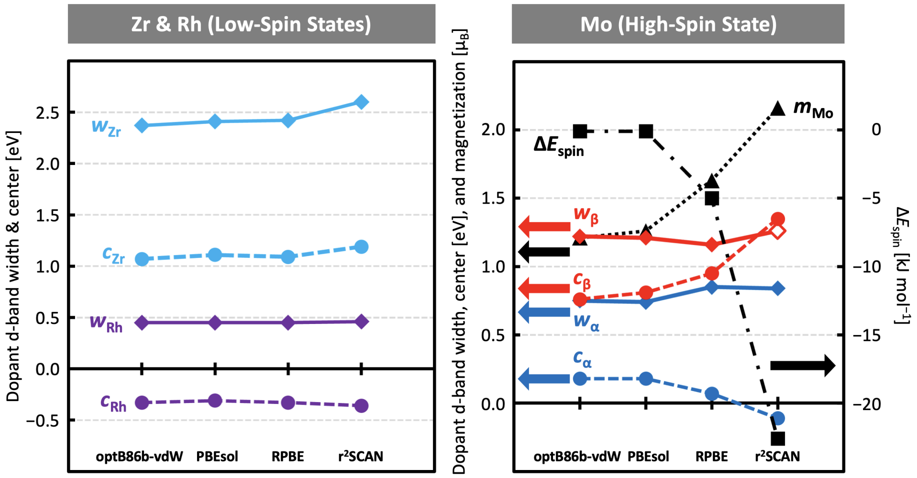


**Figure S16:** Dependence of dopant electronic structure on the choice of DFT functional, evaluated for Zr, Mo, and Rh embedded in Cu(111) using the GGA functionals optB86b-vdW, PBEsol, RPBE, and the meta-GGA r$^2$SCAN. Left: For Zr (light blue) and Rh (purple), both low-spin dopants, the $d$-band widths ($w_{\mathrm{Zr}}$, $w_{\mathrm{Rh}}$; circles) and $d$-band centers ($c_{\mathrm{Zr}}$, $c_{\mathrm{Rh}}$; diamonds) remain nearly invariant across all functionals. Right: For Mo, a high-spin dopant, both the spin moment ($m_{\mathrm{Mo}}$; black triangles) and the energetic stabilization of the high-spin state relative to the low-spin state ($\Delta E_{\mathrm{spin}}$; black squares) increase with functionals that enhance localization, notably r$^2$SCAN. The $d$-band width ($w_\alpha$, $w_\beta$; diamonds) and center ($c_\alpha$, $c_\beta$; circles) for the $\alpha$ (blue) and $\beta$ (red) spin channels of Mo, however, show only minor variation across the tested functionals. An open symbol indicates the $\beta$-spin channel $d$-band width from r$^2$SCAN, where a side peak slightly exceeding 50% of the main peak was excluded from the FWHM calculation to show the width of the main peak.

evident in the meta-GGA functional r$^2$SCAN, which partially corrects for self-interaction errors and yields a higher spin moment and a significantly larger $\Delta E_{\mathrm{spin}}$ (Table S21). However, the spin-resolved $d$-band centers ($c_\alpha$, $c_\beta$) show smaller variations than the spin magnetization and stabilization energy, and the $d$-band widths ($w_\alpha$, $w_\beta$) remain comparatively stable across all functionals, indicating that $d$-band broadening is less sensitive to changes in electron localization. These results support the robustness of the predicted $d$-band widths, even in systems with spin polarization. While spin-dependent properties may require a more careful treatment of exchange and correlation, the conclusions drawn in this work remain valid across the tested functionals. For future studies involving magnetic dopants, especially $3d$ transition metals where spin effects are likely more pronounced, we recommend the use of meta-GGA functionals such as r$^2$SCAN.

**Table S21:** Spin moment ($m_{\mathrm{Mo}}$; in $\mu_\mathrm{B}$), spin-state stabilization energy ($\Delta E_{\mathrm{spin}}$; in kJ/mol), and spin-resolved $d$-band centers ($c_\alpha$, $c_\beta$; in eV) and widths ($w_\alpha$, $w_\beta$; in eV) for Mo dopants embedded in Cu(111), calculated using four different DFT functionals: optB86b-vdW, PBEsol, RPBE, and r$^2$SCAN. Improved treatment of electron localization from GGA to meta-GGA increases both the magnetization and the stabilization of the high-spin state, while the $d$-band features, particularly the $d$-band widths, remain relatively stable across functionals.

| | optB86b-vdW | PBEsol | RPBE | r$^2$SCAN |
|---|---|---|---|---|
| $m_{\mathrm{Mo}}$ | 1.21 | 1.26 | 1.63 | 2.16 |
| $\Delta E_{\mathrm{spin}}$ | 0 | 0 | −5 | −23 |
| $c_\alpha$ | 0.18 | 0.18 | 0.07 | −0.11 |
| $c_\beta$ | 0.76 | 0.81 | 0.95 | 1.35 |
| $w_\alpha$ | 0.75 | 0.74 | 0.85 | 0.84 |
| $w_\beta$ | 1.22 | 1.21 | 1.16 | 1.26 |

# S9 Impact of Dopant $d$-Band Width Variation on Catalytic Selectivity

To investigate how variations in dopant $d$-band width influence catalytic performance, we link literature data on the selective hydrogenation of crotonaldehyde over Cu- and Ag-based SAAs[S11] with our findings on the electronic structure of the active dopant sites. This reaction can yield two partially hydrogenated products, butanal and crotyl alcohol, depending on the preferred adsorption mode of crotonaldehyde at the active site. Specifically, the molecule may adsorb either through its C=C double bond (ene) or its C=O double bond (ald), leading to distinct hydrogenation pathways.

Figure S17 illustrates the conceptual reaction network used in our analysis. A gas-phase reactant ($A_{gas}$) can adsorb onto the catalyst surface in two distinct configurations, forming adsorbed intermediates $A_1$-surface and $A_2$-surface. These intermediates then undergo surface reactions to yield different products, B and C, respectively.

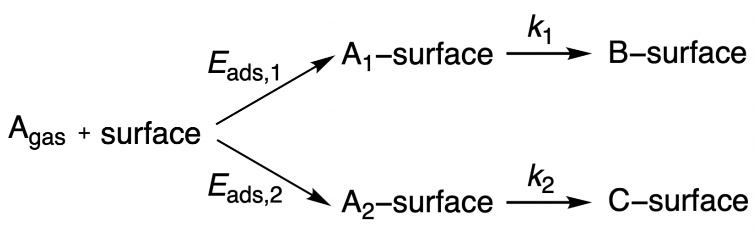


**Figure S17:** Reaction scheme for the adsorption of a reactant $A_{gas}$ from the gas phase onto a catalytically active surface, adopting two distinct adsorption motifs, $A_1$-surface and $A_2$-surface, with corresponding adsorption energies $E_{ads,1}$ and $E_{ads,2}$, respectively. Each adsorbed species subsequently undergoes a surface reaction to form different products, B and C, with respective rate constants $k_1$ and $k_2$.

Catalyst selectivity is reflected in the product distribution, which is governed by the relative rates of the two competing reaction steps. Assuming that each pathway is irreversible and follows Arrhenius kinetics, the rate constants can be expressed as:

$$k = A \cdot e^{-E_a/(RT)}, \tag{S9}$$

where $A$ is the pre-exponential factor, $E_a$ is the activation energy, R is the gas constant, and $T$ is the temperature.

Assuming identical pre-exponential factors for both reaction pathways, the ratio of products C to B becomes:

$$\frac{[\mathrm{C}]}{[\mathrm{B}]} \approx \exp\left(-\frac{E_{a,2} - E_{a,1}}{RT}\right), \tag{S10}$$

where [B] and [C] are the concentrations of the respective products, and $E_{a,2}$ and $E_{a,1}$ are the activation energies associated with the two pathways.

To connect the product ratio to adsorption properties, we assume a Brønsted–Evans–Polanyi (BEP)-type relationship that linearly relates activation energies to adsorption energies at the active site:

$$E_{a,i} \approx \beta E_{\mathrm{ads},i} + E_0 \tag{S11}$$

Here, $E_{\mathrm{ads},i}$ are the adsorption energies for the two binding motifs (ene and ald), taken from ref. S11, and $E_0$ is an offset. Assuming that both reactions follow the same BEP relation, or that the offsets $E_0$ are approximately equal, and adopting $\beta = 1.0$, consistent with ref. S14, we obtain:

$$E_{a,2} - E_{a,1} \approx E_{\mathrm{ads},2} - E_{\mathrm{ads},1} \tag{S12}$$

This expression is consistent with the expectation that, under the assumption of a constant intrinsic barrier, differences in apparent activation energies can be approximated by the corresponding differences in adsorption energies.

Substituting this into the Arrhenius expression for the product ratio yields:

$$\frac{[\mathrm{C}]}{[\mathrm{B}]} \approx \exp\left(-\frac{\beta\left(E_{\mathrm{ads},2} - E_{\mathrm{ads},1}\right)}{\mathrm{R}T}\right) \tag{S13}$$

We apply this framework to Zr and Rh dopants in Cu(111) and Ag(111) hosts using adsorption energies reported by Spivey et al.[S11] The resulting product ratios are summarized in Table S22, enabling a comparison of selectivity across different SAAs as a function of dopant electronic structure.

**Table S22:** Adsorption energies (in kJ $mol^{-1}$) for the two adsorption motifs, 'ald' and 'ene', on Zr- and Rh-doped Cu(111) and Ag(111) surfaces, adopted from ref. S11. Also shown are the differences in adsorption energies ($E_{ads}(ald) - E_{ads}(ene)$) and the predicted product ratios for crotonealdehyde hydrogenation. These ratios are estimated using an Arrhenius-type relation and assuming a linear Brønsted–Evans–Polanyi (BEP) relationship with $\beta = 1$, consistent with values reported in ref. S14. Specifically, the last column shows the relative change in product distribution upon switching from Cu to Ag as the host metal for a given dopant, indicating the sensitivity of selectivity to the host. This selectivity shift is large when the dopant $d$-band width $w_{dop}$ (in eV) changes significantly between hosts and small when it remains relatively constant. The values of $w_{dop}$ have been computed as part of this study.

| TM | surface | $w_{dop}$ | $E_{ads}(ald)$ | $E_{ads}(ene)$ | $E_{ads}(ald) - E_{ads}(ene)$ | $(\frac{[ene]}{[ald]})_{Cu}/(\frac{[ene]}{[ald]})_{Ag}$ |
|---|---|---|---|---|---|---|
| - | Cu(111) | - | −19 | −19 | 0 | - |
| - | Ag(111) | - | −22 | −22 | 0 | - |
| Zr | Cu(111) | 2.37 | −158 | −93 | 65 | 260.9 |
| Zr | Ag(111) | 1.12 | −172 | −83 | 88 | - |
| Rh | Cu(111) | 0.45 | −39 | −70 | −31 | 1.6 |
| Rh | Ag(111) | 0.39 | −52 | −82 | −29 | - |

These results illustrate a key finding: when the dopant $d$-band width varies substantially between hosts (as for Zr), catalytic selectivity is strongly affected. For Zr, the $d$-band width drops from 2.37 eV in Cu(111) to 1.12 eV in Ag(111), resulting in a 261-fold shift in the predicted product ratio. In contrast, Rh exhibits nearly unchanged $d$-band widths (0.45 to 0.39 eV), and correspondingly, only a minor shift in selectivity (factor of 1.6). It is important to note that this analysis relies on several simplifying assumptions and approximations, including the use of a BEP-type relation, the assumption that both reaction pathways follow the same BEP relation, and similar pre-exponential factors, and is not intended to be quantitatively predictive. Instead, it is meant to qualitatively illustrate how changes in the

electronic structure of the active site, specifically the dopant $d$-band width, can influence catalytic selectivity.

Crucially, the dramatic shifts in predicted product distribution are not due to changing the dopant element itself, the active site remains the same dopant in both cases, but arise from changes in the dopant's environment, specifically the host metal. It is the influence of the host on the dopant's electronic structure that drives the observed changes in adsorption energies and, consequently, in selectivity. This is further supported by the fact that crotonaldehyde exhibits nearly identical adsorption energies on pure Cu and Ag surfaces ($-19$ and $-22$ kJ mol$^{-1}$, respectively), indicating that the direct interaction of the reactant with the host metal plays a minimal role. Instead, the host influences reactivity indirectly by modifying the dopant's electronic structure.

This analysis highlights the sensitivity of catalytic behavior to the local electronic environment of the dopant and underscores the relevance of the dopant $d$-band width as a tunable parameter for controlling selectivity in such catalysts.

# References


(S1) Klimeš, J.; Bowler, D. R.; Michaelides, A. Van der Waals density functionals applied to solids. *Phys. Rev. B* **2011**, *83*, 195131.

(S2) Kresse, G.; Hafner, J. Ab initio molecular dynamics for liquid metals. *Phys. Rev. B* **1993**, *47*, 558.

(S3) Kresse, G.; Furthmüller, J. Efficiency of ab-initio total energy calculations for metals and semiconductors using a plane-wave basis set. *Comput. Mater. Sci.* **1996**, *6*, 15.

(S4) Kresse, G.; Furthmüller, J. Efficient iterative schemes for ab initio total-energy calculations using a plane-wave basis set. *Phys. Rev. B* **1996**, *54*, 11169.

(S5) Kresse, G.; Joubert, D. From ultrasoft pseudopotentials to the projector augmented-wave method. *Phys. Rev. B* **1999**, *59*, 1758.

(S6) Jain, A.; Ong, S. P.; Hautier, G.; Chen, W.; Richards, W. D.; Dacek, S.; Cholia, S.; Gunter, D.; Skinner, D.; Ceder, G.; others Commentary: The Materials Project: A materials genome approach to accelerating materials innovation. *APL Mater.* **2013**, *1*.

(S7) Alchagirov, A. B.; Perdew, J. P.; Boettger, J. C.; Albers, R.; Fiolhais, C. Reply to "Comment on 'Energy and pressure versus volume: Equations of state motivated by the stabilized jellium model'". *Phys. Rev. B* **2003**, *67*, 026103.

(S8) Maintz, S.; Deringer, V. L.; Tchougréeff, A. L.; Dronskowski, R. LOBSTER: A tool to extract chemical bonding from plane-wave based DFT. 2016.

(S9) Nelson, R.; Ertural, C.; George, J.; Deringer, V. L.; Hautier, G.; Dronskowski, R. LOBSTER: Local orbital projections, atomic charges, and chemical-bonding analysis from projector-augmented-wave-based density-functional theory. *J. Comput. Chem.* **2020**, *41*, 1931.

(S10) Greiner, M. T.; Jones, T.; Beeg, S.; Zwiener, L.; Scherzer, M.; Girgsdies, F.; Piccinin, S.; Armbrüster, M.; Knop-Gericke, A.; Schlögl, R. Free-atom-like d states in single-atom alloy catalysts. *Nat. Chem.* **2018**, *10*, 1008.

(S11) Spivey, T. D.; Holewinski, A. Selective interactions between free-atom-like d-states in single-atom alloy catalysts and near-frontier molecular orbitals. *J. Am. Chem. Soc.* **2021**, *143*, 11897.

(S12) Perdew, J. P.; Ruzsinszky, A.; Csonka, G. I.; Vydrov, O. A.; Scuseria, G. E.; Constantin, L. A.; Zhou, X.; Burke, K. Restoring the Density-Gradient Expansion for Exchange in Solids and Surfaces. *Phys. Rev. Lett.* **2008**, *100*, 136406.

(S13) Schimka, L.; Harl, J.; Stroppa, A.; Grüneis, A.; Marsman, M.; Mittendorfer, F.; Kresse, G. Accurate surface and adsorption energies from many-body perturbation theory. *Nat. Mater.* **2010**, *9*, 741.

(S14) Michaelides, A.; Liu, Z.-P.; Zhang, C.; Alavi, A.; King, D. A.; Hu, P. Identification of general linear relationships between activation energies and enthalpy changes for dissociation reactions at surfaces. *J. Am. Chem. Soc.* **2003**, *125*, 3704.
